# Near-Sensor Edge Computing System Enabled by a CMOS Compatible Photonic Integrated Circuit Platform Using Bilayer AlN/Si Waveguides

**DOI:** 10.1007/s40820-025-01743-y

**Published:** 2025-05-19

**Authors:** Zhihao Ren, Zixuan Zhang, Yangyang Zhuge, Zian Xiao, Siyu Xu, Jingkai Zhou, Chengkuo Lee

**Affiliations:** 1 Department of Electrical and Computer Engineering, National University of Singapore, Singapore, 117583 Singapore; 2 Center for Intelligent Sensors and MEMS, National University of Singapore, Singapore, 117608 Singapore; 3 National Centre for Advanced Integrated Photonics (NCAIP), Singapore, 639798 Singapore; 4 NUS Graduate School - Integrative Sciences and Engineering Programme (ISEP), National University of Singapore, 21 Lower Kent Ridge Road, Singapore, 119077 Singapore

**Keywords:** Photonic integrated circuits, Edge computing, Aluminum nitride, Neural networks, Wearable sensors

## Abstract

The rise of large-scale artificial intelligence (AI) models, such as ChatGPT, DeepSeek, and autonomous vehicle systems, has significantly advanced the boundaries of AI, enabling highly complex tasks in natural language processing, image recognition, and real-time decision-making. However, these models demand immense computational power and are often centralized, relying on cloud-based architectures with inherent limitations in latency, privacy, and energy efficiency. To address these challenges and bring AI closer to real-world applications, such as wearable health monitoring, robotics, and immersive virtual environments, innovative hardware solutions are urgently needed. This work introduces a near-sensor edge computing (NSEC) system, built on a bilayer AlN/Si waveguide platform, to provide real-time, energy-efficient AI capabilities at the edge. Leveraging the electro-optic properties of AlN microring resonators for photonic feature extraction, coupled with Si-based thermo-optic Mach–Zehnder interferometers for neural network computations, the system represents a transformative approach to AI hardware design. Demonstrated through multimodal gesture and gait analysis, the NSEC system achieves high classification accuracies of 96.77% for gestures and 98.31% for gaits, ultra-low latency (< 10 ns), and minimal energy consumption (< 0.34 pJ). This groundbreaking system bridges the gap between AI models and real-world applications, enabling efficient, privacy-preserving AI solutions for healthcare, robotics, and next-generation human–machine interfaces, marking a pivotal advancement in edge computing and AI deployment.

**Supplementary Information:**

The online version contains supplementary material available at 10.1007/s40820-025-01743-y.

## Highlights

- A novel near-sensor edge computing system integrates aluminum nitride (AlN) microrings for photonic feature extraction and Si Mach–Zehnder interferometers for photonic neural network operations, achieving real-time artificial intelligence (AI) processing.
- Demonstrates high classification accuracy (96.77% for gestures, 98.31% for gaits) with low latency (< 10 ns) and minimal energy consumption (< 0.34 pJ).
- Enables low-power, high-speed AI applications with seamless hybrid photonic-electronic integration on a bilayer AlN/Si waveguide platform.

**Supplementary Information:**

The online version contains supplementary material available at 10.1007/s40820-025-01743-y.

## Graphical abstract

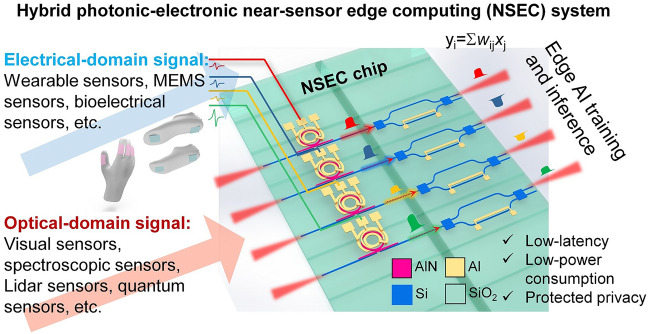

## Introduction

With the rapid development of Artificial Intelligence of Things (AIoT), the number of sensor nodes and the volume of sensing data have both increased dramatically [1]. This poses significant challenges to the computational capacities and energy consumption of artificial intelligence (AI) [2]. Recent emerging large-scale AI models have necessitated the establishment of dedicated AI data centers. These cloud-based computing frameworks are under immense pressure to manage the rising demands for data bandwidth, high transmission rates, extensive storage capacities, and efficient coding and decoding processes [3]. To address these limitations, edge computing has emerged as a complementary solution to cloud computing [4–7]. This framework reduces the volume of data transmitted to the cloud, thereby alleviating bandwidth and energy requirements. Additionally, edge computing offers enhanced privacy by enabling data processing closer to the source, using metadata, and minimizing the exposure of sensitive information. As AI systems evolve to meet the needs of modern applications, integrating edge computing with cloud infrastructures is becoming an essential strategy for achieving sustainable and efficient AI operations.

**Fig. 1:**
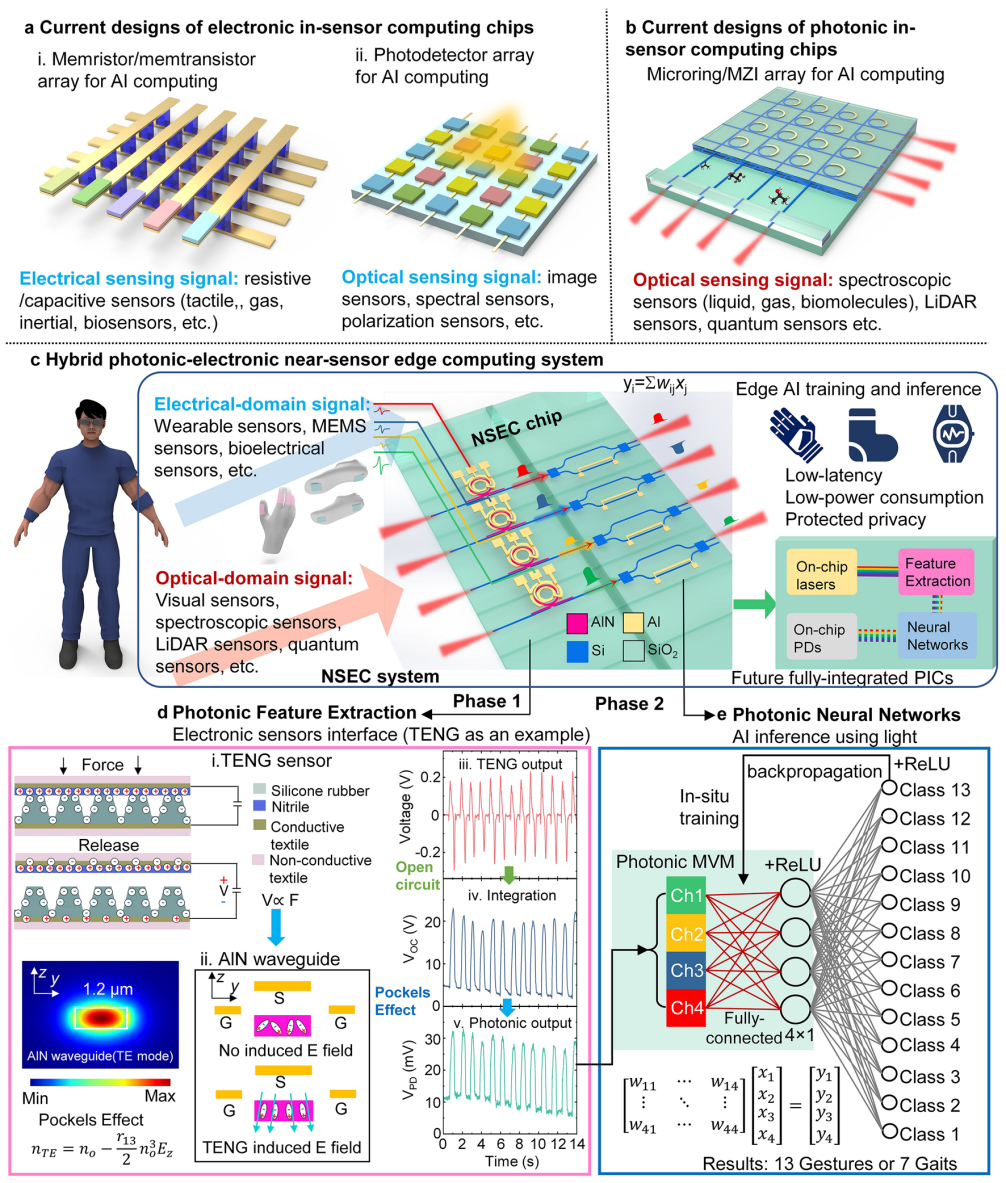
System illustration of hybrid photonic-electronic near-sensor edge computing(NSEC) enabled by AlN/Si photonic integrated circuits. **a** In-sensor computing with electronic-integrated circuits. (i) Microelectronic in-sensor computing chip enabled by memristors/memtransistors for electric sensing signal processing. (ii) Optoelectronic in-sensor computing enabled by photodetectors for optical sensing signal processing. **b** In-sensor computing with photonic integrated circuits. **c** Hybrid photonic-electronic near-sensor edge computing with AlN/Si photonic integrated circuits **d** Photonic feature extraction units on NSEC chip. (i) Schematic diagram of the TENG force/pressure sensor, where the output voltage is proportional to the applied force or pressure during the contact-release cycles of the triboelectric structures. (ii) The voltage applied to the top electrode modulates the AlN waveguide structure via the induced electric field along the z-direction, affecting the fundamental TE mode. (iii) Original TENG sensor output under repeated applied force, as measured by a conventional electric circuit. (iv) The open-circuit voltage output of the TENG under applied forces. This signal is equivalent to the mathematical integration of the original TENG sensor output due to charge accumulation in the open-circuit configuration. (v) Modulated photonic output induced by the electric field of the open-circuit ground-signal-ground (GSG) electrode on top of the AlN waveguide structure, which is directly connected to the TENG sensor. Thanks to the charge accumulation and retention mechanism of a capacitor, the photonic output reflects the time-domain profile of the applied force, effectively performing feature extraction of the TENG sensor data equivalent to an integration operation. **e** Photonic neural network unit on the NSEC chip. After photonic feature extraction, the data is fed into Si MZIs to perform photonic matrix–vector multiplication as the linear layer of the PNN. The weights are adjusted by the applied voltage to the Si MZIs. The nonlinear activation is then performed by the backend electronics, and error backpropagation is used to update the weights, achieving in-situ training

The traditional von Neumann architecture, which relies on logical operations performed by fundamental components like transistors, has served as the backbone of computing for decades. However, with the rapid expansion of AI applications, there has been a notable shift toward neuromorphic chips, such as memristors and memtransistors [8–12]. These devices enable parallel matrix operations and are better suited for the complex computational demands of AI workloads. Beyond foundational hardware upgrades, industry trends have increasingly focused on integrated solutions tailored for edge computing. Companies like STMicroelectronics have developed hybrid chips that combine microelectromechanical systems (MEMS) inertial sensors or microphones with memory, data buffers, and transmission capabilities, all packaged into a single unit. Besides, MEMS-based edge computing has demonstrated significant potential for edge AI applications by integrating sensing and computation within a single device [13–15]. Recent advancements include MEMS neural networks for direct sensor-to-computation processing and MEMS reservoir computing systems leveraging stiffness modulation for efficient, real-time data processing at the edge with 99.8% accuracy and chaos forecasting [16]. These innovations highlight the growing emphasis on compact, efficient systems that minimize the need for external data processing and storage. In the research domain, in-sensor computing has emerged as a key area of focus [17, 18]. This approach integrates sensing and computation directly at the hardware level, offering significant advantages in terms of latency and energy efficiency. As shown in Fig. 1a, examples include purely electronic systems that pair resistive-based sensors with memristors, as well as optoelectronic frameworks utilizing programmable photodetectors [19–22]. By processing data directly at the sensor, in-sensor computing minimizes reliance on centralized systems, marking a significant shift toward next-generation AI architectures that are faster, more efficient, and optimized for edge applications. This localized preprocessing at the sensor level effectively reduces data volume, transmission load, and overall energy consumption, enabling faster and more efficient AI computations. Various works on in-sensor computing have been published to handle images [19–32], gas [33, 34], or biological sensing information [35–40].

Mennel et al. demonstrate in-sensor image recognition in a two-dimensional (2D) photodiode array by utilizing the tunable photoresponsivity matrix as a synaptic weight matrix so that the processed image data is computed during the photodetection process [30]. Zhou et al. present a neuromorphic event-based image sensor that leverages a spiking neural network for in-sensor processing [25]. This innovation reduces redundant data generation during sensing and eliminates the necessity for data transfer between sensors and computational units, thereby significantly improving efficiency and processing speed. Furthermore, Chen et al. introduce an all-analog chip, denoted as ACCEL, which integrates electronics and optics [23]. This chip utilizes free-space diffractive optical computing as an optical encoder for feature extraction. Then the classification tasks are achieved directly using weighted photodetectors arrays with embedded analog circuits, eliminating the need for analog-to-digital converters. This approach results in a remarkably low computing latency of 72 ns per frame, showing the advances in using edge computing to process image data. In addition to image processing, Li et al. proposed a groundbreaking "perception-memory" system that seamlessly integrates electronic tattoos with memristors, showcasing a highly energy-efficient and real-time biosignal processing approach [38]. This innovative work not only advances wearable AI systems but also enhances user interaction capabilities, paving the way for next-generation smart healthcare and human–machine interfaces. Currently, in-sensor and near-sensor computing primarily rely on microelectronic or optoelectronic devices. To achieve low latency and low-power edge computing, further advancements are needed in device integration and computational capacities.

Photonic integrated circuits (PICs) [41–43] have garnered extensive application in the realm of AI accelerators [43–48], attributable to their elevated integration levels, high-dimensional parallel computational capabilities, and broad bandwidth characteristics. Diverging from signal crosstalk encountered in electrical circuits, light of different wavelengths within the same optical path can be independently extracted through wavelength demultiplexing for photodetection [49–52]. Consequently, photons of distinct wavelengths can carry disparate streams of information and concurrently transmit in parallel through the same medium [53–55]. Govern by the photonic memory [56–58] enabled by phase change materials (PCMs), Dong et al. demonstrate a high-dimensional in-memory photonic tensor core that leverages spatial, radio frequency, and optical wavelength domains, increasing parallelism of data processing by a factor of 25, significantly enhancing performance [59]. Xu et al. proposed and developed Taichi, which is a large-scale photonic chiplet system with an integrated diffractive-interference hybrid design and distributed computing architecture, achieving 160 TOPS/W energy efficiency, and significant improvements in AI-generated content efficiency [60]. Furthermore, PICs have emerged as a transformative platform for in-sensor computing (Fig. 1b), enabling advanced computational capabilities directly at the sensor level using silicon photonic waveguides or on-chip photodetector [61–63]. Liu et al. [63] demonstrate an energy-efficient on-chip waveguide-based neuromorphic in-sensor computing solution using a responsivity-tunable graphene photodetector integrated with silicon waveguides, enabling multimodal data processing, including image preprocessing, gesture recognition, and spectroscopic classification in the mid-infrared range. Xiao et al. [62] present a photonic in-sensor computing system that processes optical-domain spectroscopy sensing signals using a silicon photonic processor, achieving 97.58% accuracy in classifying 45 protein classes and enabling efficient multimodal sensory data processing.

In the broader context of edge AI, two primary frameworks “wireless-driven AI architectures and fiber-to-the-home (FTTH) infrastructures” offer substantial opportunities for advancing edge computing systems. These frameworks underscore the potential for entirely optical edge computing, where data is processed exclusively in the optical domain, effectively overcoming many limitations of traditional electronic systems, such as latency and energy inefficiency. However, a significant challenge remains in bridging optical edge computing systems with electrical-domain signals, particularly for wearable sensors and other electronic devices that generate non-optical outputs. Our research addresses this challenge by proposing a novel framework for integrating electrical-domain sensor signals into an optical edge computing system. This approach enables efficient processing of electrical signals within the optical domain, creating a seamless interface between electronic sensors and photonic computing systems. Moreover, this framework paves the way for future enhancements that include direct processing of optical-domain signals from applications such as virtual reality (VR) and augmented reality (AR), which require high-speed, low-latency data handling [64, 65]. For instance, the system connects directly to wearable sensors, converting their electrical signals into optical formats for processing within the photonic edge computing framework. This capability ensures compatibility with current sensor technologies while establishing a scalable platform for next-generation applications. The integration of both electrical and optical signals within a unified edge computing architecture reduces latency, enhances computational efficiency, and supports a diverse range of use cases. By enabling a fully integrated optical edge computing platform with input terminals of electrical and optical signals, this research advances the field of edge AI, providing a versatile and high-performance solution for emerging applications interfacing with FTTH infrastructures. The proposed system bridges critical gaps between electronic and photonic technologies, aligning with the evolving demands of AI-driven innovations and setting the stage for next-generation architectures that are faster, more efficient, and highly adaptable.

## Experimental Section

### Design of Hybrid Photonic-Electronic Near-Sensor Edge Computing System

A novel near-sensor edge computing (NSEC) system is proposed, uniquely integrating two input modalities, electrical signals from wearable sensors and optical signals for advanced applications, on a unified platform, as shown in Fig. 1c. This dual-modality design eliminates the need for multiple data conversions, enabling seamless on-chip processing of both electrical and optical inputs. By leveraging photonic feature extraction for electrical signals and neural network computations for optical data, the NSEC system achieves significant reductions in energy consumption, latency, and storage requirements. This innovative approach not only enhances the versatility of edge AI architectures but also offers a scalable solution for integrating multimodal inputs, paving the way for advanced applications such as wearable sensor networks, AR, and VR. The NSEC chip, which is the key component of the NSEC system, is designed with two main functional blocks: photonic feature extraction and photonic neural networks, which correspond to the two key phases of AI edge computing. In phase 1, aluminum nitride (AlN) microring resonators (MRRs) convert signals from a variety of wearable electronic IoT sensors into optical signals by electro-optic (EO) conversion aid by Pockels effect, simultaneously extracting features during this conversion (Fig. 1d). In phase 2, Si Mach–Zehnder interferometers (MZIs) perform photonic neural network computations by thermo-optic effect on the extracted optical signals, resulting in the desired AI processing outcomes (Fig. 1e).

The feature extraction process leverages the unique EO properties of AlN MRRs, which enable efficient modulation of optical signals in response to voltage inputs. AlN, a wide-bandgap piezoelectric material, exhibits exceptional EO performance due to the Pockels effect, allowing its refractive index to change linearly with the applied electric field [66, 67]. This property provides a high-speed and energy-efficient platform for directly interfacing with electrical-domain signals. In this proof-of-concept demonstration, the triboelectric nanogenerator (TENG) serves as a self-powered input sensor, generating voltage signals proportional to the applied pressure or force during its contact-separation process (Fig. 1d–i). The AlN MRRs capitalize on the TENG-generated voltage to modulate optical signals in real time. The capacitive nature of the TENG and AlN system ensures compatibility with the open-circuit configuration, allowing charge accumulation without the need for external power sources. This makes AlN an ideal interface material for converting electrical-domain signals from various sensors into the optical domain. While the TENG is employed here as a demonstration, the AlN-based system is not limited to this sensor type. Its compatibility extends to a variety of wearable sensors and MEMS devices, such as resistive pressure sensors, accelerometers, and bioelectrical sensors, enabling seamless integration with a broad range of edge AI applications.

The superior EO properties of AlN ensure low latency, high modulation bandwidth, and minimal power consumption, making it a robust interface between electrical-domain sensing signals and optical-domain processing. This versatility positions AlN as a critical enabler for photonic edge computing systems designed to process data from diverse sensor modalities efficiently and accurately. The voltage signals from TENG sensors induce an electric field along the z-axis of the AlN waveguide, which in turn modulates its refractive index via the Pockels effect (Fig. 1d-ii). We design the length (1.2 µm) and width (0.5 µm) of the AlN waveguide to support the fundamental transverse electric (TE) mode. As shown in Fig. 1d-ii, the refractive index of the AlN waveguide under the TE mode and the electric field intensity along the z-axis can be expressed by the following equation:

1 $${n}_{\rm TE}={n}_{\rm o}-\frac{{r}_{13}}{2}{n}_{\rm o}^{3}{E}_{\rm z}$$

where *n*_o_, *r*_13_, *E*_z_ is the refractive index of ordinary light, electro-optic coefficient, and the electric field intensity. This modulation results in a resonance wavelength shift in the AlN MRR, correlating the optical output with the input electrical signals from the TENG sensor (Fig. 1d-ii-v). By fixing the wavelength at the resonant peak, the optical output signal reflects the applied pressure or force, allowing for dynamic and accurate feature extraction in the time domain. The original TENG output typically consists of a positive spike during the contact process and a negative spike during the release process (Fig. 1d-iii). This output captures only the changes in applied force, rather than its continuous temporal profile, making it insufficient for distinguishing dynamic actions such as stepwise bending of a glove sensor versus distinct, separate bending motions. By integrating the TENG signal through the AlN MRR, our system converts this spike-based data into a continuous signal that aligns with the applied force in the time domain (Fig. 1d-iv, v). This feature extraction process is mathematically equivalent to performing an integration operation on the original TENG data, enabling the retention of complete temporal dynamics. This mathematical integration ensures that the collected output signal faithfully represents the continuous pressure or force applied, enabling precise and dynamic feature extraction. Unlike traditional readout circuits that rely on instantaneous current release, our photonic feature extraction provides a seamless mapping of the sensor input to its dynamic temporal profile. Our previous studies confirm that this integration-based feature extraction approach not only enhances the accuracy of neural network classification but also supports real-time, continuous monitoring of sensor inputs [68–70]. By preserving the temporal fidelity of the signal, the photonic neural network can effectively analyze dynamic inputs, ensuring accurate and efficient signal processing for advanced sensing applications.

After feature extraction, the processed optical data is transmitted to the Si waveguide layer and directed to Si MZIs (Fig. 1e), where it undergoes weighting matrix manipulation as part of a fully connected 4 × 4 photonic neural network (PNN). The weights in the network are dynamically adjusted in situ by applying voltage to the MZIs. Specifically, a TiN heater is used to locally heat one arm of the MZI, inducing a temperature difference between the two arms. This creates a refractive index difference, resulting in a phase shift at the output ports. The optical intensity at one output port varies based on the constructive or destructive interference caused by this phase difference. The weighted optical signals are then summed using a combiner and measured by a photodetector for further processing. This system supports in-situ training by leveraging backpropagation. During the training phase, the nonlinear activation (e.g., ReLU) and backpropagation are performed by backend electronics. The errors are propagated back to update the MZI weights, enabling in-situ training and real-time adaptability. After training, the NSEC chip operates independently, handling AI inference tasks entirely on-chip. The photonic PNN computations are integrated seamlessly with backend electronics, ensuring efficient weight updates and robust processing. In the proof-of-concept demonstration presented in this paper, the size of the input and output neurons is set to 4. However, the system is inherently scalable, allowing the size of the network to be increased linearly by incorporating additional hybrid photonic-electric NSEC building blocks. This scalability enables the system to support more sensor nodes and handle larger numbers of input and output channels, making it highly adaptable for broader applications. Additionally, the in-situ training capability enhances the system’s ability to learn and adapt to specific applications, making it suitable for complex tasks requiring continuous updates and optimization.

### Fabrication and Characterization of Bilayer AlN/Si Photonic Integrated Circuits

The NSEC chip fabrication process is intricately designed and executed on the bilayer AlN/Si waveguide platform within the advanced facilities of the 8-inch photonic foundry at Advanced Micro Foundry Pte Ltd in Singapore. This process follows a series of precise steps to ensure the creation of a photonic chip for NSEC applications (Detailed process flow is shown in Fig. S2). The fabrication starts with the utilization of a standard Silicon-On-Insulator (SOI) wafer featuring a 220 nm device layer. This foundational layer sets the stage for subsequent processes aimed at defining the critical components of the chip. To define the Si waveguide, the wafer undergoes deep ultraviolet (DUV) lithography and precise Si etching steps. After this step, the fabrication process proceeds with the growth of a SiO_2_ layer using plasma-enhanced chemical vapor deposition (PECVD), employing Tetraethyl orthosilicate (TEOS) as the precursor material. To ensure a uniformly flat surface conducive to subsequent depositions and processes, the wafer undergoes chemical mechanical planarization (CMP). The next stage involves the deposition of AlN using physical vapor deposition (PVD). A subsequent round of DUV lithography and precise etching is employed to define the AlN waveguide accurately, ensuring its alignment with the Si waveguide for efficient light propagation and modulation. Continuing the process, TEOS SiO_2_ deposition and CMP processes are repeated to create additional insulating layers and maintain the desired surface flatness. Following this, DUV lithography and etching tools are utilized once again to define the SiO_2_ pattern, creating a distinct height difference essential for the electrode arrangement of the AlN MRR. The final stages of the fabrication process involve the deposition and etching of TiN and Al layers separately. These layers are meticulously crafted to form the microheater for Si thermo-optic devices and the electrode for the AlN MRR, respectively. Additionally, another layer of SiO_2_ is deposited to facilitate the creation of electrical interconnections, essential for the overall functionality and integration into larger systems.

**Fig. 2:**
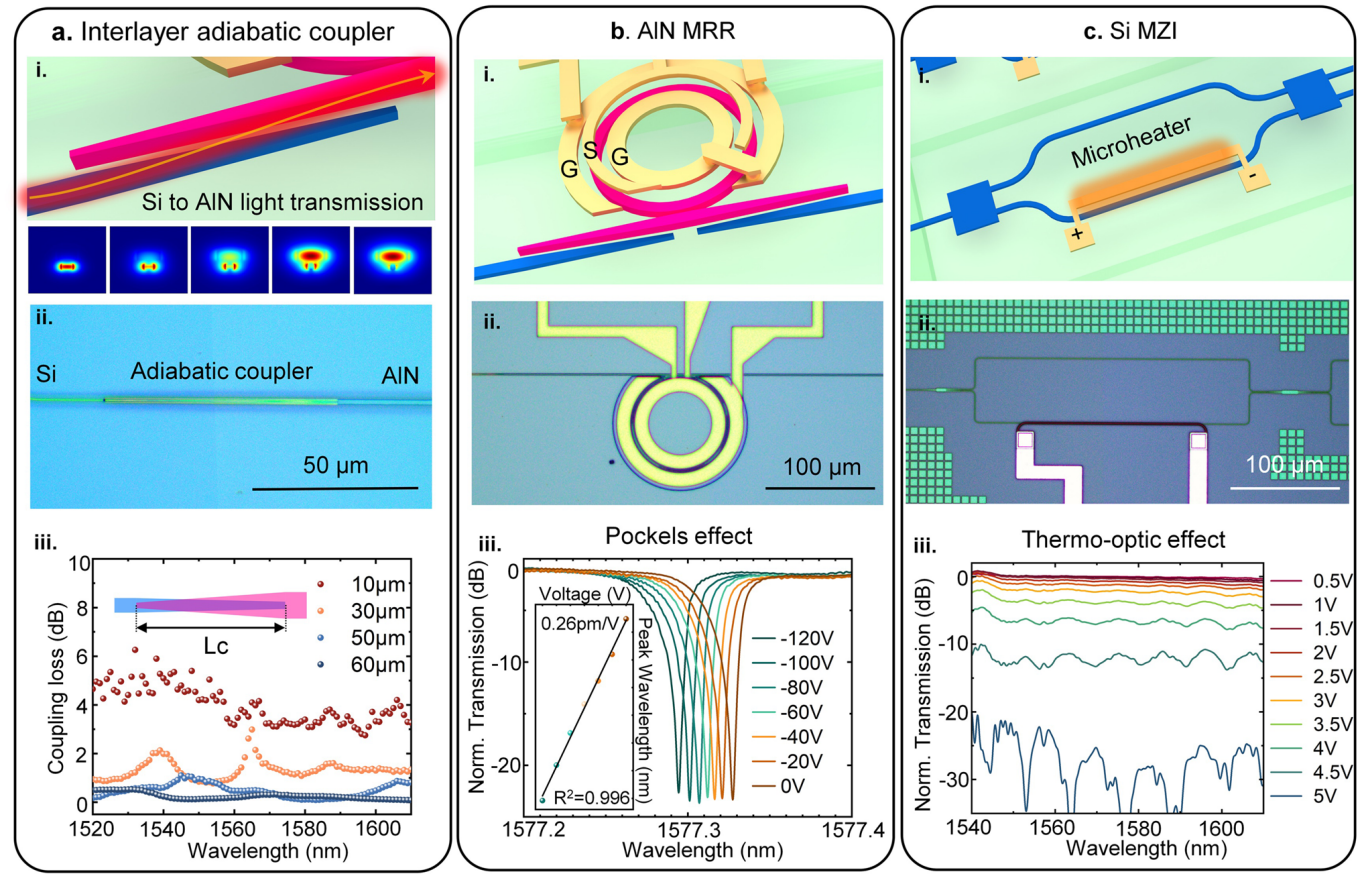
Characterization of photonic devices on NSEC chip. **a** Interlayer coupler of Si/AlN dual-layer photonic waveguide. (i) Schematic diagram of the interlayer adiabatic coupler, demonstrating light coupling from the Si waveguide layer (bottom) to the AlN waveguide layer (top). The inset shows the electric field profile at various regions along the coupler, illustrating the progression of light coupling. (ii) Optical microscope image of the adiabatic coupler. iii. Measured coupling loss across the full spectrum of the telecommunication band, indicating ultra-broadband low-loss operation of the interlayer adiabatic coupler. **b** AlN electro-optic microring resonator. (i) Schematic diagram of the bilayer AlN MRR, highlighting the modulation mechanism based on the Pockels effect in AlN. (ii) The optical microscope image of the AlN MRR (iii) Measured tuning properties of the AlN MRR, showcasing the electro-optic modulation performance enabled by the Pockels effect. **c** Si thermo-optic MZI. (i) Schematic diagram of the Si MZI, illustrating its role in performing the weighting function in the neural. (ii) The optical microscope image of the Si MZI. (iii) Measured MZI spectra under different applied voltages, demonstrating the ability to control signal weights through voltage adjustments

The NSEC photonic chip leverages a bilayer waveguide structure consisting of a Si bottom layer and an AlN top layer, fabricated via monolithic integration. Efficient light coupling between these layers is achieved using interlayer adiabatic couplers (Fig. 2a), designed to minimize optical loss during transitions. The coupling mechanism is illustrated schematically in Fig. 2a-i, showcasing the gradual transfer of light from the Si waveguide to the AlN waveguide. The electric field profile along various regions of the coupler highlights the progression of coupling. The fabricated adiabatic coupler, shown in Fig. 2a-ii, achieves an ultra-broadband low-loss performance with a coupling loss of just 0.04 dB per transition, as measured across the telecommunication band (Fig. 2a-iii). The AlN electro-optic modulators operate on the Pockels effect, allowing dynamic modulation of optical signals in response to applied voltages. The bilayer AlN MRR is depicted schematically in Fig. 2b-i, and the microscope image of the fabricated device is shown in Fig. 2b-ii. Characterization results presented in Fig. 2b-iii reveal a resonance tuning property of 0.26 pm V^−1^ under direct current (DC) conditions, demonstrating robust electro-optic modulation performance. These capabilities enable precise photonic feature extraction, seamlessly translating TENG sensing signals into the optical domain. The schematic structure and optical image of Si MZIs for photonic neural network computations are shown in Fig. 2c-i, ii. The Si MZIs execute the weighting functions required for neural network operations by inducing refractive index changes through localized heating. This mechanism adjusts the phase difference between the interferometer arms, producing constructive or destructive interference at the output ports. The measured MZI spectra under varying applied voltages (Fig. 2c-iii) demonstrate precise control over signal weights, essential for performing matrix–vector multiplication in neural network computations. Furthermore, Fig. 2c-iii highlights the modulation depth of approximately 30 dB with a half-wave voltage (*V*_π_​) of around 5.6 V, showcasing the precise thermo-optic modulation capabilities of the Si MZI across the telecommunications C and L bands. The comprehensive characterization of the interlayer coupler, AlN MRRs, and Si MZIs validates the functionality and integration of the NSEC photonic chip. More detailed design and characterization of Si/AlN dual-layer photonic device are shown in Figs. S3–S7.

### System Configuration and Characterization

The optical measurement of the NSEC chip is conducted by our customized NIR fiber-optic alignment systems. The NIR light emits from a tunable laser (Daylight 8160B lightwave measurement system) and is coupled to the NIR optical fibers (Thorlab SMF-28). The 6-axis manual alignment stages (Kouzu GXM07S) are used to align the NSEC chip and optical fibers to make the NIR light coupled to a photonic waveguide and transmit back to the output optical fibers. Photodetectors (Thorlab DET08CFC) are used to collect the optical signal and convert to electrical signal. The wearable electric sensor signal is connected to AlN MRRs for photonic feature extraction and the weighting signal is added by electrical circuits for photonic AI computing. Then the output optical signal is fed into photodetectors to transfer optical signals to electric signals for analysis. For the electrical circuits, the microcontroller (STMicroelectronics, NUCLEO-F746ZG) was used to control the 12-bit ADC board (MAX22531EVKIT) to collect the analog output from the amplifying circuit after the photodetector. The microcontroller also controls the DAC board with 12-bit accuracy (MAX11300PMB1) to apply the voltage.

### Implementation of Neural Networks in NSEC System

The TENG sensor dataset is collected from the NSEC chip to achieve in-situ training and photonic inference. In the data collection process, the participants were asked to wear TENG gloves and TENG socks to repeat the hand gestures and normal walking status. Finally, a total of 40 samples (80%) were used as the training set, while the other 10 samples (20%) were used as the testing set. The development of a robust gesture recognition system involved the training of a two-layer Multilayer Perceptron (MLP) on a standard consumer-grade computer. The MLP architecture, implemented using Python with a PyTorch backend, comprised two fully connected layers capable of extracting intricate patterns from the four-channel sensor data. The ultimate objective was to accurately predict the 13 different hand gestures based on the input sensor data. During the training phase, the network was optimized using the cross-entropy loss function, aiming to minimize the discrepancy between the predicted logits and the actual target values. To facilitate efficient optimization, an adaptive moment estimation (Adam) optimizer was employed, leveraging a learning rate of 0.0001. This approach allowed the network to dynamically adjust its learning rate based on the gradient of the loss function, enhancing convergence and overall training efficiency. The training process was conducted over 400 epochs, enabling the network to iteratively learn and refine its predictive capabilities (Detailed loss curve is shown in Fig. S9). Each epoch involved forward and backward propagation, where the network's parameters were updated using the Adam optimizer to improve prediction accuracy. This iterative training process played a crucial role in enhancing the model's ability to generalize and make accurate predictions on unseen data. Following the completion of the training phase, the MLP model underwent rigorous testing using a dedicated testing dataset. The inference process involved feeding the testing data into the trained model to evaluate its performance in real-world scenarios. By comparing the model's predictions with the ground truth labels in the testing set, we were able to assess the accuracy and robustness of the gesture recognition system.

## Results and Discussion

### Gesture Recognition by Sensor Gloves Using Photonic Feature Extraction (Phase 1)

**Fig. 3:**
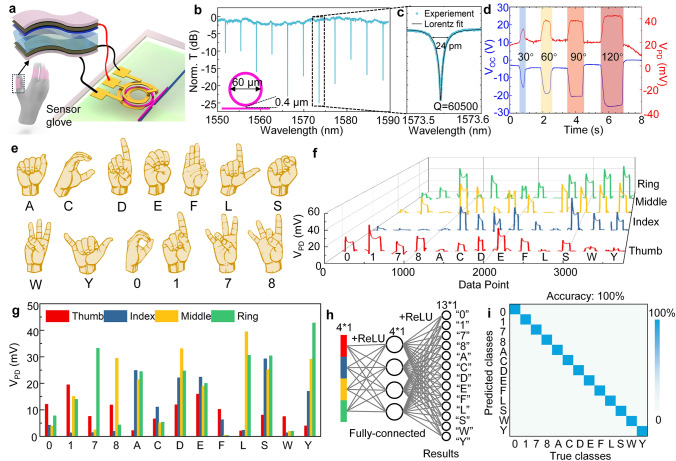
Feature extraction of TENG glove sensor using photonic chips for gesture recognition. **a** System schematic of TENG glove with pressure sensors on 4 fingers (thumb, index, middle, ring). Each sensor on different fingers is connected to AlN MRRs respectively. **b** Broadband spectrum of AlN MRR with a diameter of 60 µm and gap of 0.4 µm. **c** Zoom-in view of one resonance peak at 1577.525 nm showing a Q factor of 65700. **d** The open circuit voltage of TENG sensors and related photodetector voltage of modulated AlN MRR at 1577.526 nm wavelength for index finger bending at 30°, 60°, 90°, and 120° in continuous time response. The index finger returns to its initial straightened state after each bend. **e** 13 different gestures represent the English letters and Arabic numerals in American sign language (ASL). **f** The sensor response of 13 gestures after feature extraction on AlN MRR. **g** The extracted voltage change value of 13 different gestures. **h.** The neural network model of gesture recognition. **i** The confusion matrix of recognition results using photonic feature extraction.

The self-powered pressure sensor, utilizing TENG technology, is strategically installed on the four finger joints of a glove to capture bending motions (Fig. 3a). During finger bending, the positive and negative triboelectric materials (nitrile and silicone rubber) come into contact and generate electrical charges through friction. For photonic feature extraction of these TENG-generated signals, we employed an AlN MRR with a diameter of 60 µm and a coupling gap of 0.4 µm. The AlN MRR spectrum, presented in Fig. 3b, c, features a resonance peak at 1577.525 nm with a high Q-factor of 65,700, reflecting its superior sensitivity and precision. The signal from the index finger was directly connected to the electrodes of the AlN MRR to monitor bending-induced variations. To simultaneously measure the TENG sensor’s output voltage and the modulated optical signal, we employed an electrometer (Keithley 6514) in parallel to record the open-circuit voltage as a reference. Experimental results (Fig. 3d) illustrate four consecutive bending tests of the index finger at incremental angles of 30°, 60°, 90°, and 120°. Both the TENG output voltage and the optical output of the AlN MRR increased progressively with larger bending angles. Furthermore, the optical signal demonstrated precise temporal alignment with the electrical signal, validating the system's capability for real-time sensing and processing. To assess repeatability, the system underwent 100 consecutive 30° bending tests of the index finger. The results revealed consistent changes in both the electrical output voltage and the optical signal, with minor deviations attributed to manual control considered as system errors for neural network training. These findings underscore the robustness of AlN MRRs in performing photonic feature extraction for TENG-based pressure sensors.

Furthermore, we extended the application of photonics feature extraction from the aluminum nitride-integrated circuit to gesture recognition in American Sign Language (ASL). As illustrated in Fig. 3e, we selected 13 representative gestures, symbolizing English letters and Arabic numerals, for demonstration purposes. The signals from the TENG sensors on the four fingers were individually connected to four identical AlN MRRs for the retrieval of feature-extracted signals. One set of signals for the 13 gestures is displayed in Fig. 3f. Throughout the varying gestures, we observed a relatively stable maximum signal output, which correlates with the degree of finger bending. Specifically, we extracted the output variation values for each finger's corresponding sensor, as shown in Fig. 3g. These signals represent the input to the neural network after completing photonics feature extraction. To train the neural network model, we conducted 50 repeated tests for each gesture set, randomly dividing them into 80% for training and 20% for testing purposes. The classification task for gestures was accomplished using a fully connected neural network, and its framework is depicted in Fig. 3h. We input the signals obtained after photonics feature extraction into the trained neural network, achieving a recognition accuracy of 100% (Fig. 3i). In the subsequent sections, we will delve further into the on-chip implementation of photonics feature extraction and neural network computations, specifically focusing on photonic near-sensor edge computing.

### Gait Analysis by Sensor Socks Using Photonic Feature Extraction (Phase 1)

**Fig. 4:**
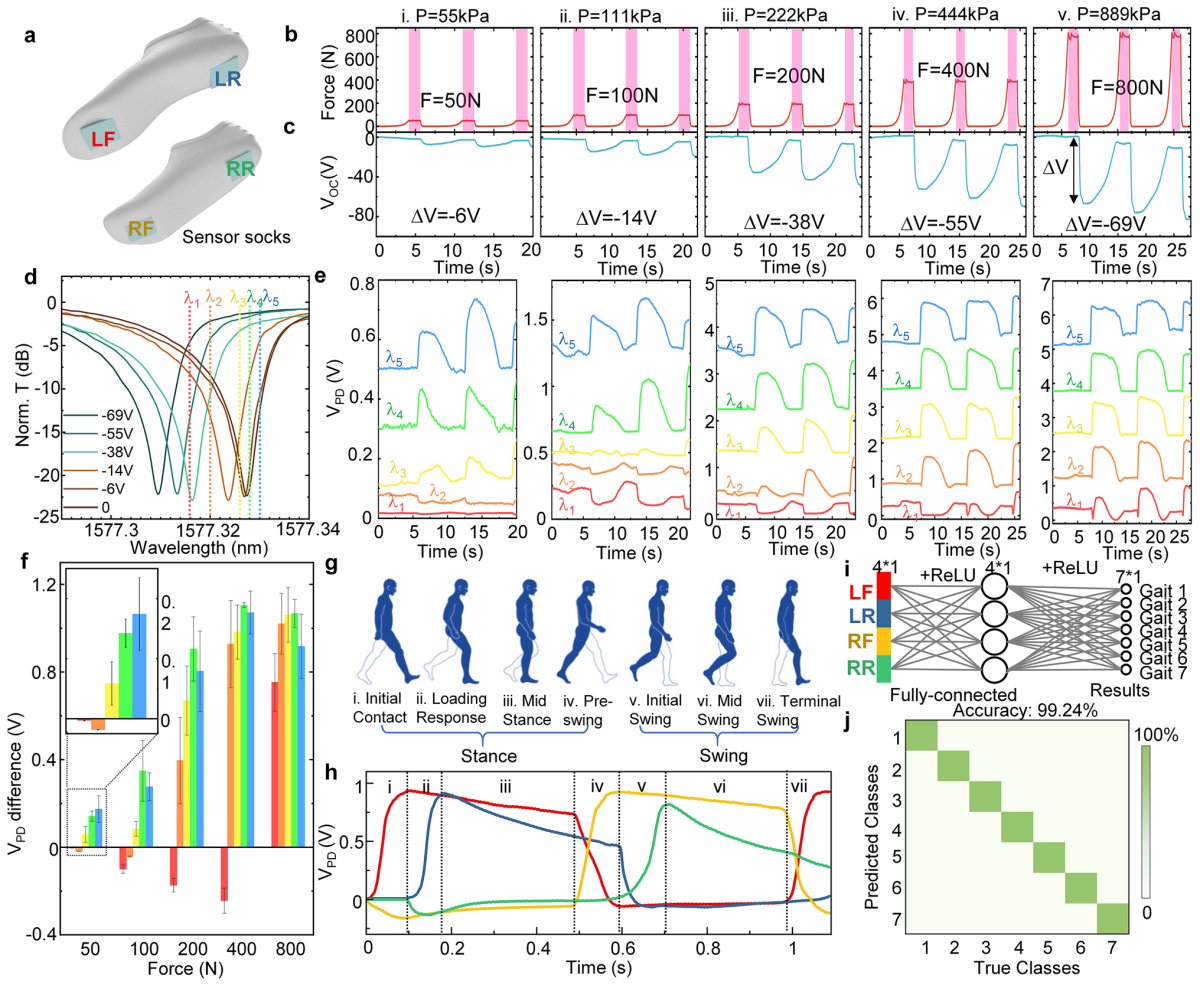
Feature extraction of TENG sock sensor using photonic chips for gait analysis. **a** schematic drawing of TENG sock sensors. 4 sensors are placed on different positions of a pair of socks-left front (LF), left rear (LR), right front (LF), right rear (RR). **b, c** Standard force measurement using TENG sock sensors. Force profile generated by force gauge at different time (**b**). Corresponding TENG sensor output measured in open-circuit condition (**c**). **d** MRR resonance spectrum at extracted voltages of TENG output. Five probing wavelengths for continuous-time monitoring are labeled accordingly. **e** Continuous-time monitoring of MRR signal corresponding to the TENG output in (**c**) at different probing wavelengths. **f** The extracted MRR signal change (photo voltage difference) at applied forces with different probing wavelengths. **g** Gait analysis states in one gait cycle. **h** The corresponding MRR signals of 4 TENG sock sensors in one gait cycle. **i** The neural network model for gait analysis using TENG sock sensors with feature extraction from MRR. **j** The confusion matrix of gait analysis state using MRR signals

In addition to the glove sensors, we have also integrated self-powered TENG sensors into socks, as illustrated in Fig. 4a. Four TENG sensors are strategically placed on the forefoot and heel of both the left and right feet, each measuring 3 × 3 cm^2^. Before conducting human gait analysis, we characterized the TENG sensors using a standard force gauge. The results, shown in Fig. 4b, c, were obtained by testing pressures of 50, 100, 200, 400, and 800 N, with three repeated measurements for each pressure condition. As the pressure increases from 50 to 800 N, the open-circuit output voltage of the TENG gradually changes from −6 to −69 V. This output is considerably larger compared to the TENG glove, necessitating the selection of an appropriate probe wavelength to read this signal variation from the AlN electro-optic microring resonator. Consequently, we tested the resonance spectra of the AlN MRR at these five output voltages, as depicted in Fig. 4d. The maximum blue-shift range of the spectrum reaches 18 pm. To observe the variation in the modulated light signal, we selected five wavelengths (λ_1_:1577.316 nm, λ_2_:1577.32 nm, λ_3_:1577.326 nm, λ_4_:1577.328 nm, and λ_5_:1577.33 nm), and the results are presented in Fig. 4e. Regardless of the wavelength, the temporal signal at each wavelength corresponds well with the variation in our input pressure signal. To better discern the patterns of change, we plotted the voltage variations in Fig. 4f. When the probe wavelength is smaller than the resonant wavelength, we observe that the modulated light signal initially decreases with voltage before reversing and increasing. This change occurs because of the blue shift in the resonance peak. Initially, the signal moves toward the valley of the resonance, but then it returns to a point of higher light intensity after passing through the valley. Conversely, when the probe wavelength is larger than the resonant wavelength, the modulated light signal consistently increases with voltage but quickly reaches saturation. After careful consideration, we selected the resonant wavelength (λ_3_:1577.326 nm) as the photonics feature for the TENG socks in the application of gait recognition. Upon ascertaining the probe wavelength, we leverage it for the recognition of human gait. The depiction of the correlation between gait recognition states and an individual's steps is presented in the illustrated Fig. 4g. Within a single gait cycle, segmentation occurs into two main phases: stance and swing, consisting of seven distinct states. The sensor socks integrate four sensors with each connected to AlN electro-optic microring resonators. Unlike the static gesture recognition discussed earlier, the sensor sock signals enable real-time dynamic monitoring. As an individual walks, these signals undergo photonics feature extraction and computational processing, resulting in classification outcomes that accurately represent the state of gait analysis. The temporal signals for the four TENG sensor channels after photonics feature extraction are shown in Fig. 4h. Through the identification of temporal markers corresponding to the elevation and grounding of the left and right feet, the dataset is labeled into seven classes, each aligning with one of the seven gait states. The data after photonics feature extraction is subsequently subjected to neural network computations, with the neural network architecture depicted in Fig. 4i. Following the training regimen, the accuracy of gait recognition attains a level of 99%, as shown in the confusion map in Fig. 4j.

### Near-Sensor Edge Computing by Photonic Feature Extraction and PNNs (Phase 2)

**Fig. 5:**
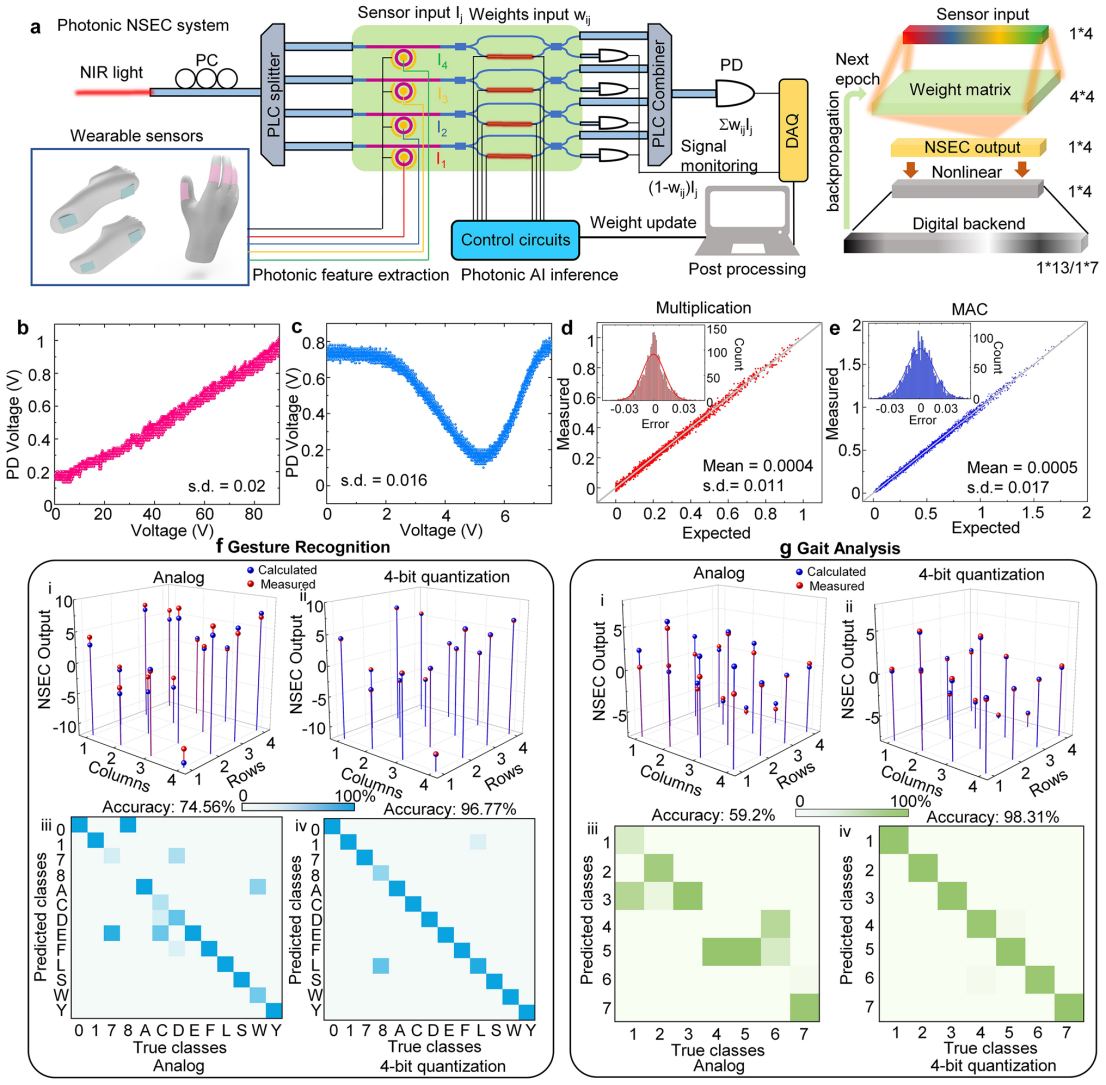
Demonstration of an NSEC system using AlN/Si PIC systems. **a** Optical characterization setup of AlN/Si photonic integrated circuit systems for NSEC. The implementation of a fully connected 4 by 4 neural network using 4 AlN MRRs for photonic feature extraction and 4 Si MZIs for matrix–vector manipulation. The right part shows the equivalent neural network model for the gesture identification and gait analysis tasks achieved by NSEC. **b** The measured modulation signal of AlN MRRs under continuous voltage sweep. The inset optical microscope image shows the device of AlN MRR and the adiabatic coupler for light transmission from the bottom Si layer to the top AlN layer. **c** The measured modulation signal of Si MZIs under continuous voltage sweeps. The inset optical microscope image shows the device of Si MZIs with a microheater. **d, e** The measured calculation accuracy of multiplication (**d**) and MAC operation (**e**) using MRR and MZI devices. **f, g** Photonic feature extraction and AI computation results of gesture recognition (**f**) and gait analysis (**g**) tasks. (i, ii) the matrix–vector manipulation results from the NSEC chip with analog operation (i) and 4-bit quantization (ii). (iii, iv) The confusion matrix of recognition results using NSEC output with analog operation (iii) and 4-bit quantization (iv)

The schematic representation of the photonic near-sensor edge computing chip system is delineated in Fig. 5a. In order to concurrently process signals from four distinct channels, a laser beam undergoes splitting through a PLC splitter, resulting in its distribution into four distinct paths. Subsequently, these separated light beams are guided into four distinct AlN electro-optic microring resonators, where sensor signals are intricately interfaced to enable preliminary photonics feature extraction. In the NSEC, the signals undergoing photonic feature extraction are seamlessly transmitted to a Si MZI for subsequent engagement in PNN computations. The modulation of the weights of PNN is orchestrated through the judicious application of voltage to the thermo-optic modulator. The modulated signals, imbued with weighted significance, are then amalgamated leveraging a PLC combiner. The calculated results are recorded through a photodetector to convert to electric signals for post-processing. In the equivalent neural network, the sensor signals, having undergone photonics feature extraction through aluminum nitride microring resonators, serve as inputs to a 4 × 4 fully connected neural network. Matrix computations are executed through silicon MZI for network output generation. Subsequently, the output optical signal is converted into a digital signal using a photodetector for backend nonlinear activation and classification operations by the digital electronic processor. During the training process, the computed loss function is iteratively updated through gradient backpropagation to adjust weights for subsequent epochs, striving for optimal accuracy. In the inference process, sensor signals are fed into the trained weight matrix. At this point, the bar state of MZI is utilized for weight matrix computation. However, owing to the presence of modulation noise in the photonic chip, computed results may exhibit deviations, thereby impacting the final accuracy of inference. Simultaneously, monitoring signals from the cross-state of MZI are also captured. While distinct from the bar state, these signals exhibit complementary relationships. Mathematically, the response to the modulation power can be conceptualized as two sinusoidal waves with a phase difference of 90 degrees. In other words, if the bar state output is represented as *w*_*ij*_*I*_*j*_, the cross-state can be considered as (1 − *w*_*ij*_)*I*_*j*_. The signals from the four channels of the cross-state are not aggregated, serving the purpose of monitoring the accuracy of each weight. Real-time adjustments to the applied voltage of MZI are made to correct discrepancies. In addition to hardware-level optimizations, algorithmic enhancements are pursued through the quantization of weights. However, prior to specific algorithmic optimizations, understanding the noise characteristics of the entire system is crucial. Therefore, characterization of noise in both the AlN MRR and the Si MZI is undertaken.

In Fig. 5b, the modulation characteristics and noise of the aluminum nitride electro-optic modulator at a fixed wavelength are presented. The optical microscope image of the aluminum nitride electro-optic modulator used in the tests is displayed in the inset of Fig. 5b. Additionally, we have zoomed in to showcase the adiabatic coupler section of the waveguide, a critical component for guiding light from the bottom silicon layer to the top aluminum nitride layer. The test wavelength is fixed at the peak wavelength of the microring resonator (1573.325 nm), and a cyclic scan of the voltage is conducted from 1 to 90 V with intervals of 0.2 V. After 20 cycles of testing, the results are plotted in Fig. 5b. It is evident that as the voltage increases, the optical signal also increases while maintaining a favorable linearity. The standard deviation during the aluminum nitride electro-optic modulator process is calculated to be 0.02. Maintaining the same wavelength, we continue to assess the noise of the silicon thermo-optic modulator, as depicted in Fig. 5c. However, when applying an electrical signal, the microheater on one arm of the MZI heats, leading to a change in the refractive index of that arm and, consequently, a variation in output. We scan the voltage from 0 to 7.5 V with intervals of 0.05 V, detecting the output in the bar state. From Fig. 5c, it can be observed that the optical signal initially decreases and then increases with the rising voltage. This behavior is attributed to the fact that the modulation of the MZI is effectively induced by phase, thus exhibiting periodicity. The output signal follows a sinusoidal relationship with the phase, resembling a sine wave. The refractive index change caused by the thermo-optic modulator is directly proportional to the temperature. Hence, the output optical signal should exhibit a sinusoidal relationship with the power of the modulating electrical signal, specifically a sinusoidal relationship with the square of the voltage. Therefore, we choose the modulation signal before the π phase, i.e., before V_π_ = 5.2 V, for the weight update function. We repeat the voltage cycle 20 times to characterize the noise of the Si MZI, resulting in a final standard deviation of 0.016.

Upon individually characterizing the noise of the AlN MRRs and Si MZIs, we seamlessly integrate these devices to examine the noise distribution during the computational process. Commencing with the selection of 1000 sets of random numbers as multiplication factors, two numbers are chosen randomly for multiplication, and the numerically computed result is denoted as 'expected'. Simultaneously, these numerical values are translated into corresponding electrical modulation signals and fed into the aluminum nitride microring resonator and Si MZI. The optical responses are then measured and normalized to align with the numerically calculated outcomes. Statistical analysis reveals a Gaussian noise distribution in the results of all random number multiplications, with a standard deviation of 0.011, as illustrated in Fig. 5d. Subsequently, we assess the accuracy of both multiplication and addition operations, where the results of two sets of multiplications are summed. In the optical computation, these results are presented in Fig. 5e, highlighting that the noise adheres to a Gaussian distribution, with a standard deviation of 0.017. Following the characterization of computational noise, we showcase near-sensor edge computing through demonstrations of gesture recognition and gait analysis. Initiating the training process with analog weights for sensor gloves and socks signals, we perform feature extraction by connecting TENG sensors to AlN MRR. Neural network computations are then executed directly in Si MZI. The computational outcomes once converted to digital signals, undergo nonlinear activation and loss function calculation on a computer. Subsequent backpropagation through gradient descent updates the Si MZI signals for subsequent epochs, steadily converging the loss function toward zero. Post-training, we evaluate the precision of each weight through the cross-state of Si MZI, maintaining signals with identical intensity for the four AlN MRRs. Under analog conditions, the measured weight values exhibit some deviation from the actual measured values (Fig. 5e, f–i), influencing the precision of subsequent neural network computations. Consequently, we propose a quantization approach for weight assignment. This method involves fixed numerical values for each weight state, with the difference between adjacent weights determined by the quantized number of bits. When noise is smaller than the difference between two quantized weights, the input weights are likely to fall into the correct state, minimizing overall error and enhancing accuracy. Simulation results demonstrate that when weights are quantized to 4 bits, neural network accuracy surpasses 96%. While increasing bit numbers further enhances accuracy, a trade-off between bit numbers and noise is necessary. Notably, the standard deviation of the multiplication operation primarily falls within the range of ± 0.033, closely approaching the spacing between states of a 4-bit system. Consequently, with 4-bit quantization, the majority of weights (> ~ 99%) align within an accurate range, yielding high neural network accuracy. Having validated the quantization weight algorithm, we proceed to conduct real-time on-chip analyses of signals from TENG gloves and socks. Gesture recognition accuracy is reported as 74.56% (analog weight) and 96.77% (4-bit quantization weight), while real-time gait analysis accuracy stands at 59.2% (analog weight) and 98.31% (4-bit quantization weight). These results collectively affirm the viability of our comprehensive near-sensor edge computing system for AIoT applications.

### Near-Sensor Edge Computing for Metaverse Applications

**Fig. 6:**
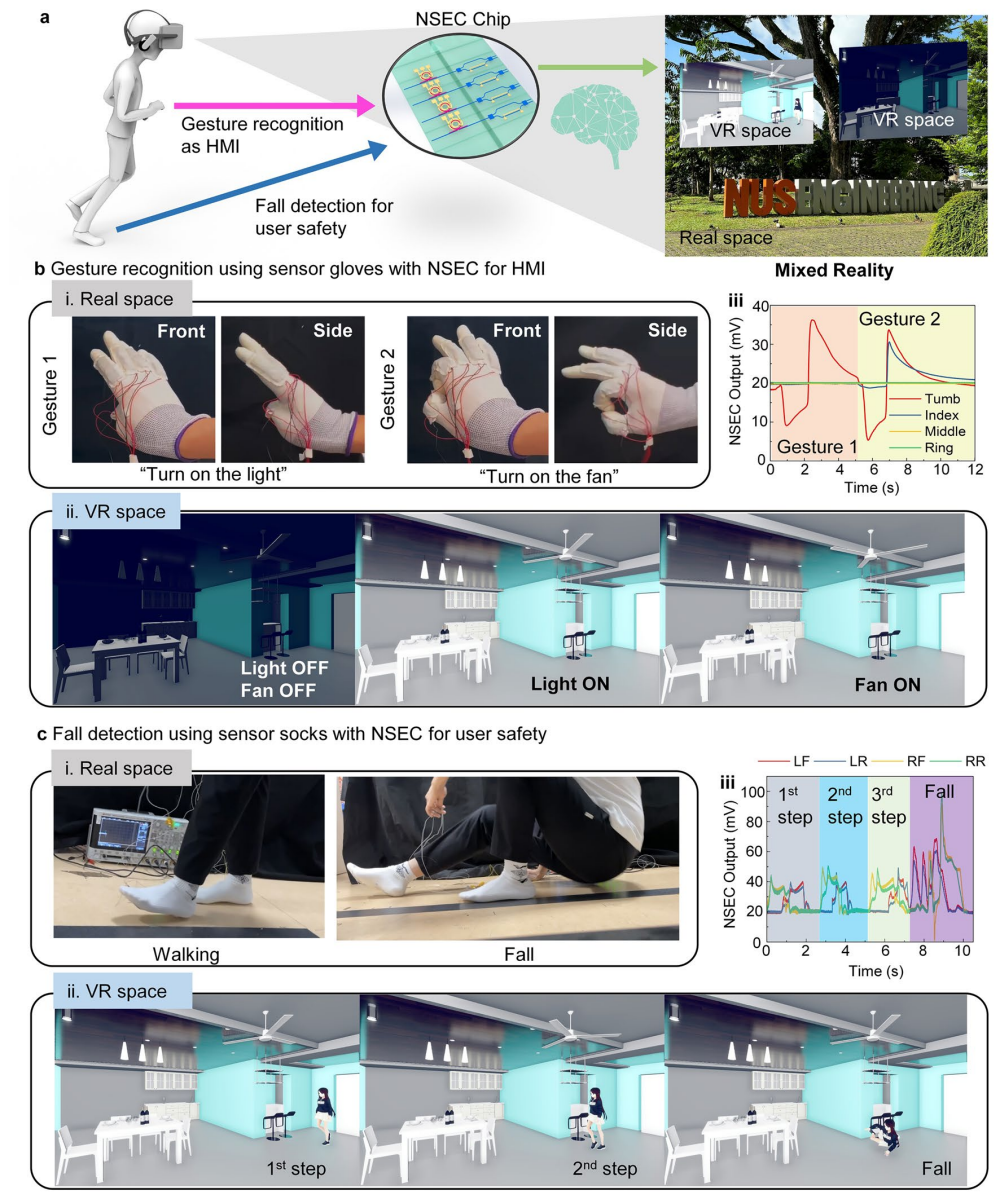
Near-sensor edge computing toward metaverse applications. **a** Schematic drawing of the intended use case for mixed reality applications assisted by sensor gloves and socks with NSEC chip. **b** The sensor gloves provide the human–machine interface with machine learning results generated by NSEC. (i) The gesture photo in real space. (ii) The corresponding control in VR space with the gestures. (iii) The NSEC output for different gestures. **c** The sensor socks provide user safety monitoring with gait analysis results generated by NSEC in a mixed reality environment. (i) The gait patterns in real space. (ii) The corresponding gait state is demonstrated by an avatar in VR space. (iii) The NSEC output for different gait states

In recent years, with the rapid rise of AIoT, the metaverse has emerged as a popular application direction, gradually changing people's lifestyles. Recently, the introduction of next-generation smart devices such as Apple Vision Pro has accelerated the empowerment of entering the metaverse world for individuals. In mixed reality (MR) applications, gesture recognition has become a fundamental human–computer interaction mode for controlling virtual space interfaces. Therefore, developing a gesture recognition human–machine interface with low data volume, low latency, and low-power consumption has become an important development trend. On the other hand, due to the overlap of VR space and display space in mixed reality, users may encounter obstructed lines of sight and risks of falling while moving in physical space. Although MR equipment like Apple Vision Pro currently limits users from high-speed movement, detecting accidental falls is also crucial for user safety. Based on these two demands, our developed near-sensor edge computing system paired with sensor gloves and socks can effectively address these issues (Fig. 6a).

Firstly, the sensor gloves can directly track the degree of finger bending of the user and quickly obtain control signals for controlling VR space through low latency, low-power AI processing by NSEC. This significantly reduces the data volume at the sensing end and the computational load of the neural network compared to methods that rely on video/image or light detection and ranging (LiDAR)-based hand motion recognition, thereby greatly reducing data latency and power consumption. As shown in Fig. 6b, we perform two gestures in physical space, representing "turn on the lights" and "turn on the fan" in virtual space. Through computational processing by NSEC, we achieve a real-time and efficient human–machine interface, displaying the corresponding "turn on the lights" and "turn on the fan" commands operated by the user in VR space. The corresponding NSEC signals are displayed in Fig. 6b-iii. Furthermore, the sensor socks can monitor the user's gait characteristics in real-time, and through machine learning computations by NSEC, it can detect falls by users. As shown in Fig. 6c, we demonstrate normal walking and falling by the user in physical space. The output signal from NSEC allows the avatar created in VR space to simulate the user's gait in real-time and detect instances of the user falling, issuing warnings promptly. The corresponding NSEC signals are displayed in Fig. 6c-iii.

Through these two application demos, we observe that utilizing near-sensor edge computing in combination with various modal sensors provides a solution with low data volume, low latency, and low-power consumption compared to traditional methods relying on video or image recognition and LiDAR signal recognition. This solution can be widely applied in various AIoT fields such as human–machine interface, health monitoring, smart home devices, and more, thereby offering a promising approach for metaverse applications.

## Conclusions

The hybrid photonic-electronic NSEC system represents a pioneering integration of high-speed photonic computing chips with wearable sensors, marking a paradigm shift in edge AI applications interfacing with FTTH infrastructures. By seamlessly combining AlN electro-optic MRRs for photonic feature extraction and Si thermo-optic MZIs for PNN computations, the NSEC chip achieves real-time AI processing with minimal latency, low-power consumption, and high classification accuracy. This innovation highlights the transformative potential of photonic computing in wearable AI systems. By integrating TENG sensors, the NSEC chip demonstrates its capability to operate in a nearly energy-neutral manner, powered entirely by the sensor's friction-induced charge generation. This eliminates the need for external power sources, making it highly efficient for pressure sensing and AI processing applications. The NSEC chip realized by the bilayer AlN/Si photonic platform ensures low energy consumption, as the AI computations are performed directly at the edge, with peak energy consumption during inference maintained at just 0.34 pJ. Additionally, the high modulation bandwidth (> 10 GHz [67, 71]) of the AlN electro-optic modulators facilitates ultra-low latency processing (< 0.1 ns), enabling rapid signal transmission and real-time AI inference. The overall AI latency of the NSEC system is approximately 10 ns, which includes the photodetector response (~ 0.2 ns) and nonlinear activation processing by the FPGA (~ 10 ns), ensuring highly efficient edge AI computations. This integrated architecture eliminates reliance on cloud-based data transmission, significantly reducing data latency, improving energy efficiency, and enhancing privacy—key advantages that align with the advancing trends of AI toward agentic AI and physical AI at the edge. A distinguishing feature of the NSEC system is its ability to seamlessly integrate two input modalities: electrical-domain signals and optical-domain signals. By supporting electrical inputs from wearable sensors, such as resistive sensors [72–74], MEMS sensors [75–77], triboelectric sensors [78–82], and bioelectrical sensors [83, 84], alongside optical inputs from devices like spectroscopic sensors [85–91], visual sensors [24], LiDAR [92], and polarization detectors [93–95], the system demonstrates unprecedented versatility. This dual-modality capability enables efficient data processing across a wide range of sensor types, bridging the gap between electrical and optical sensing technologies within a unified photonic computing platform.

The NSEC system has been validated through real-world applications, including static gesture recognition using sensor gloves and dynamic gait analysis with sensor socks, achieving high classification accuracies exceeding 96%. These results underscore its capability for advanced applications such as healthcare monitoring, smart wearable systems, and metaverse interfaces, which demand high-speed, low latency, and energy-efficient data processing. Furthermore, the NSEC system sets the stage for integrating additional wearable sensors with photonic computing chips, creating comprehensive edge AI systems. This transformative approach represents a paradigm shift in how wearable AI systems process data by embedding intelligence directly within physical devices. By addressing key challenges, including data latency, energy consumption, and privacy concerns, the NSEC system establishes a robust foundation for decentralized, high-performance, and energy-efficient edge AI systems across diverse applications.

## Supplementary Information

Below is the link to the electronic supplementary material.

Supplementary file1 (DOCX 6719 KB)
