# Supplementary material for: Near-Sensor Edge Computing System Enabled by a CMOS Compatible Photonic Integrated Circuit Platform Using Bilayer AlN/Si Waveguides
Source: Nanomicro Lett. 2025 May 19;17:261. doi: 10.1007/s40820-025-01743-y (PMC12089552; doi:10.1007/s40820-025-01743-y)
Supplement: Supplementary file 1 — Supplementary file1 (DOCX 6719 KB) [file 40820_2025_1743_MOESM1_ESM.docx]

Supporting Information for

**Near-Sensor Edge Computing System Enabled by a CMOS Compatible Photonic Integrated Circuit Platform Using Bilayer AlN/Si Waveguides**

Zhihao Ren^1,2,3^, Zixuan Zhang^1,2,^ Yangyang Zhuge^1,2^, Zian Xiao^1,2^, Siyu Xu^1,2^, Jingkai Zhou^1,2^ and Chengkuo Lee^1,2,3,4,*^

^1^Department of Electrical and Computer Engineering, National University of Singapore, 117583, Singapore

^2^Center for Intelligent Sensors and MEMS, National University of Singapore, 117608, Singapore

^3^ National Centre for Advanced Integrated Photonics (NCAIP), 639798, Singapore

^4^ NUS Graduate School - Integrative Sciences and Engineering Programme (ISEP), National University of Singapore, 21 Lower Kent Ridge Road, Singapore 119077, Singapore.

*Corresponding author. E-mail: [elelc@nus.edu.sg](mailto:elelc@nus.edu.sg) (Chengkuo Lee)

Supplementary Text

**Note S1 Propagation loss of AlN and Si waveguide**

We designed the AlN and Si waveguides to operate in the fundamental TE mode and minimize loss. As shown in **Fig. S3a**, the thicknesses of the AlN and Si waveguides are 0.4 and 0.22 µm, respectively. By adjusting the waveguide widths, we explored the propagation modes and equivalent refractive indices, as depicted in **Fig. S3b, c**. For the AlN waveguide, a width of 1 µm corresponds to the fundamental TE mode. We chose this width as the basic design for the AlN layer waveguide device. As for the Si waveguide, we selected a width of 0.45 µm as the basic design for the waveguide device.

To characterize the transmission loss of the fabricated AlN and Si waveguides, we employed the cut-back method. By normalizing the insertion loss of spiral waveguides of different lengths, we eliminated fiber coupling loss to calculate the waveguide propagation loss, as shown in **Fig. S3d, e**. We measured a transmission loss of 4.4 dB/cm for the AlN layer and 1.4 dB/cm for the Si layer.

Despite the relatively high transmission loss of the AlN waveguide, our bilayer structural design allows light to propagate mostly through the Si waveguide, entering the AlN layer only at the AlN electro-optic modulator (EOM) for electro-optic modulation. The modulated light signal is then routed back to the Si waveguide layer, reducing the overall transmission loss. The advantage of this bilayer platform lies in its effective utilization of electro-optic modulation properties of AlN while mitigating its higher transmission loss.

**Note S2 Design and characterization of AlN/Si interlayer coupler**

An essential component in our bilayer waveguide platform is the interlayer coupler, which facilitates the coupling of optical signals between different layers, such as from Si to AlN or vice versa. Among various types of interlayer couplers, the adiabatic coupler stands out due to its efficiency in mode transition between waveguides with different effective indices. This transition is achieved by varying the widths of the waveguides in each layer, thereby adjusting the effective indices of the waveguide modes. For instance, in the case of coupling from a Si waveguide to an AlN waveguide, the width of the Si waveguide decreases gradually to reduce its effective index *n_eff_*), while the width of the AlN waveguide increases to raise its effective index. This design ensures that the optical mode transitions smoothly from the waveguide with a lower effective index to the one with a higher effective index. As illustrated in **Fig. S4a**, the width of the Si waveguide narrows while the width of the AlN waveguide broadens. It's crucial to maintain these waveguides in their fundamental transverse electric (TE) mode throughout the transition because most of the waveguide device in the NSEC chip is designed in fundamental TE mode. The region where these two adiabatic waveguides overlap is termed the coupling length, denoted as *Lc*. If *Lc* is too short, the mode transition gradient becomes steep, leading to higher losses. Conversely, an excessively long *Lc* increases the device footprint, which can negatively impact the overall performance of the photonic integrated circuit.

Based on the calculated effective indices for the fundamental TE modes of AlN and Si waveguides, we have designed the width profiles of the adiabatic coupler (**Fig. S4b**). The Si waveguide width varies from 0.45 µm to 0.2 µm, and the AlN waveguide width varies from 0.45 µm to 1 µm. As light propagates through the adiabatic coupler, the *n_eff_* of the waveguides cross over, ensuring a smooth transition from the Si to the AlN layer. We also calculated the coupling losses for different *Lc* values (**Fig. S4c**). Our simulations show that coupling losses decrease as *Lc* increases. However, to balance loss reduction and device footprint, we selected an *Lc* of 60 µm for use in the NSEC chip design.

In Finite-Difference Time-Domain (FDTD) simulations, only mode transition losses are considered because the waveguide surfaces are assumed to be perfectly smooth. In practice, however, the waveguide surfaces exhibit some roughness due to the etching process, which introduces additional scattering losses. These losses are more pronounced when *Lc* is shorter. Mode analysis of different cross-sections of the adiabatic coupler (**Fig. S4d**) demonstrates the gradual transition of the waveguide mode from the Si layer (bottom) to the AlN layer (top). This detailed analysis ensures that our adiabatic coupler design facilitates efficient and low-loss mode coupling between the Si and AlN waveguides, essential for the performance of the bilayer platform in the NSEC chip.

**Note S3 Characterization of coupling and crossing loss in AlN/Si bilayer platform**

To accurately characterize the interlayer coupling loss and crossing loss on the AlN-Si photonic platform, we developed a test kit with multiple couplers on a waveguide. As shown in **Fig. S5a**, the test kit includes waveguides of the same total length but with varying numbers of adiabatic couplers. The normalized transmission for these configurations is plotted, and by performing a linear fit, we determined the coupling loss to be 0.12 dB per coupler. This measurement method effectively eliminates the insertion and propagation loss of the waveguide and, by accumulating the losses from multiple couplers, enhances the signal-to-noise ratio, allowing precise calculation of the small loss associated with each coupler. In practical devices, such as the hybrid AlN/Si microring, the total loss for light coupling-out of and into the Si layer is 0.24 dB. Although this represents an additional loss compared to devices operating solely in the AlN layer, it is mitigated by the higher propagation loss in AlN waveguides (4.4 dB/cm) compared to Si waveguides (1.4 dB/cm). Therefore, for photonic integrated circuits (PICs) with light propagation longer than 800 µm, the bilayer structure provides a lower overall optical loss than a single AlN layer, highlighting the advantage of the bilayer structure for complex PIC applications.

We also characterized the crossing loss, which occurs when Si and AlN waveguides overlap, causing some loss due to evanescent field coupling. As shown in **Fig. S5b**, we measured the normalized transmission for waveguides with varying numbers of crossings. Linear fitting of these data yielded a crossing loss of 0.04 dB per crossing. It is important to note that crossing loss, unlike coupling loss, can be minimized through careful layout design to avoid unnecessary crossings of Si and AlN waveguides. In the NSEC application demonstrated in this study, no Si-AlN waveguide crossings were conducted to achieve the AI computing system. However, as the complexity of PIC systems increases, such crossings may become unavoidable. Nevertheless, their impact on the total loss can be minimized and is generally negligible.

**Note S4 Characterization of AlN/Si bilayer microring resonators**

We first investigated the passive performance of AlN/Si bilayer MRR by fabricating and experimentally characterizing devices with various dimensions. **Fig. S6a** outlines our primary modifications to three critical parameters: the MRR radius(*R*), the *gap* between the MRR and the bus waveguide, and the length (*Lc*) of the interlayer adiabatic coupler, which is distinctive to bilayer AlN-Si waveguide structure. The experimental results are illustrated in **Figs. S6b-e**, with coupling gaps, varied from 400 to 600 nm. Specifically, **Figs. S6, c** display results for a fixed radius of 30 μm and coupling lengths of 50 and 75 μm, respectively, while **Figs. S6d, e** show results for a radius of 50 μm with the same coupling lengths of 50 and 75 μm.

The data demonstrate that the coupling gap profoundly affects the coupling strength of the MRR. As the gap changes from 600 to 400 nm, the MRR transitions from an over-coupled to a critically coupled state, resulting in enhanced resonance strength, as evidenced by a higher extinction ratio of the resonance peaks. However, the quality factor (Q) slightly decreases due to increased radiation losses. By comparing **Figs. S6b, d** with **Figs.** **S6c, e**, respectively, we observe that an increase in radius corresponds to a decrease in the free spectral range (FSR) of the MRR. Additionally, a comparison between **Figs. S6b, c** as well as **Figs. S6d, e**, indicates that larger *Lc* enhances signal stability of MRR spectra. These findings underscore the critical role of geometric parameters in tuning MRR performance on the AlN/Si platform. The ability to control the coupling gap, MRR radius, and coupling length allows for precise adjustments to resonance characteristics, including extinction ratio, Q factor, and FSR. This tunability is essential for optimizing PICs across various applications.

Furthermore, we conducted a detailed characterization of AlN electro-optic modulators (EOMs) with two distinct electrode configurations, as depicted in Fig. S6. In configuration V1, the electrode intersects the waveguide, while in configuration V2, it does not (**Figs. S7a, b**). The performance of these EOMs with different electrode designs is comprehensively illustrated in **Figs. S7c-f**. The modulation response of both V1 and V2 configurations under an applied DC bias was thoroughly examined, with results presented in **Figs. S7g, h**. Our findings indicate that the V2 configuration exhibits superior tunability, demonstrating a wavelength shift of 30 pm at an applied voltage of 200 V.

**Supplementary Figures**


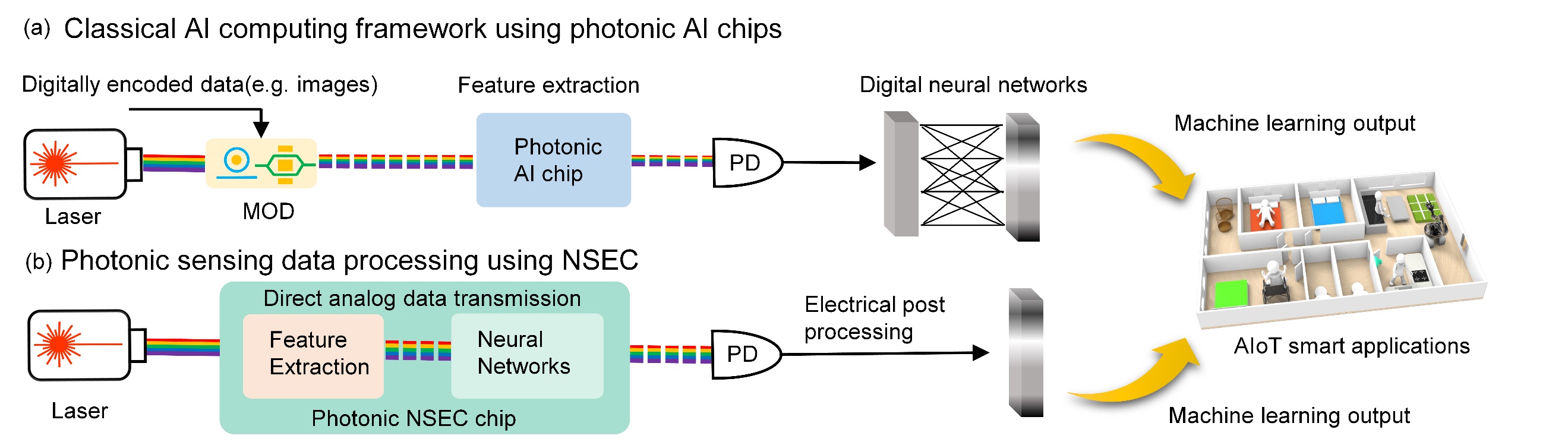


**Fig. S1** System framework of NSEC system. (**a**) Classical AI computing framework using photonic AI chips. (**b**) Photonic sensing data processing using photonic neural networks with hybrid photonic-electric NSEC system

**
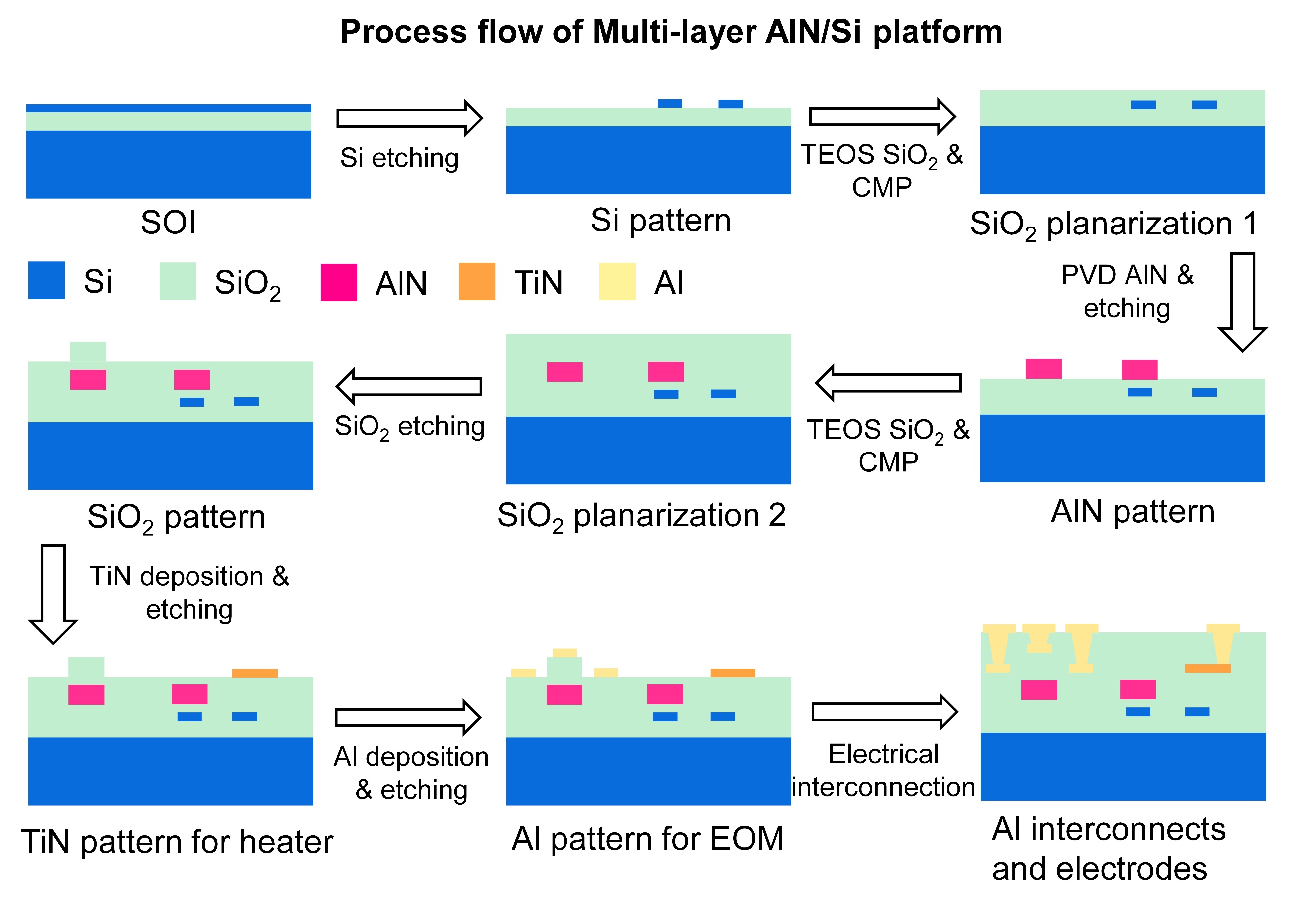
**

Fig. S2 The fabrication process flow of bilayer AlN/Si waveguide platform


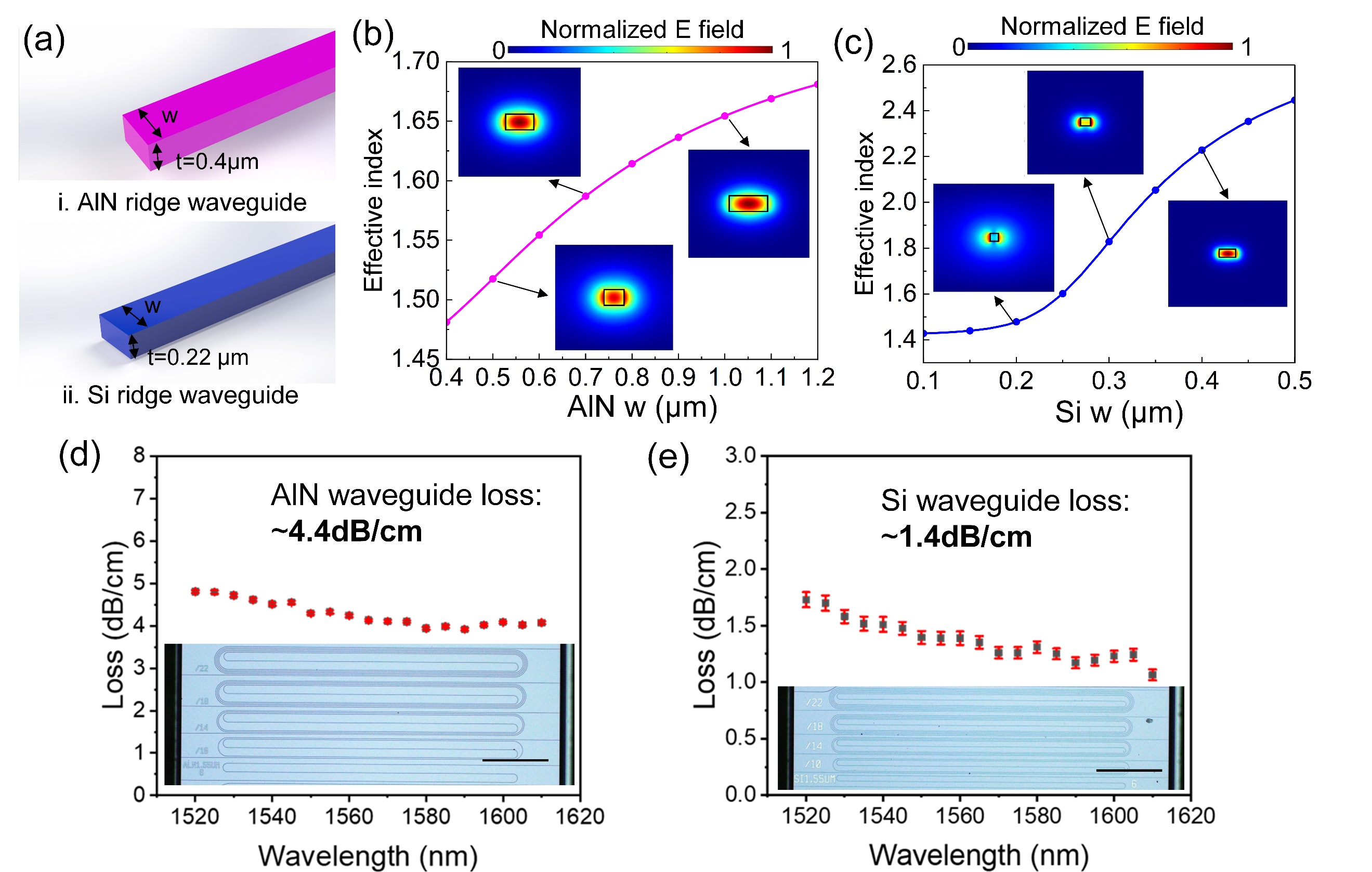


Fig. S3 Characterization of propagation loss of AlN and Si waveguide used in NSEC chip. (a) schematic diagram of AlN(i) and Si(ii) waveguides. (b, c) the simulated effective index and mode profile(electric field) of AlN (b) and Si (c) waveguide with different widths. (d, e) measured propagation loss of AlN (d) and Si (e) waveguide at wavelength range from 1520 nm to 1620 nm.

The inset optical microscope images show the spiral waveguides for propagation loss measurement using the cut-back method


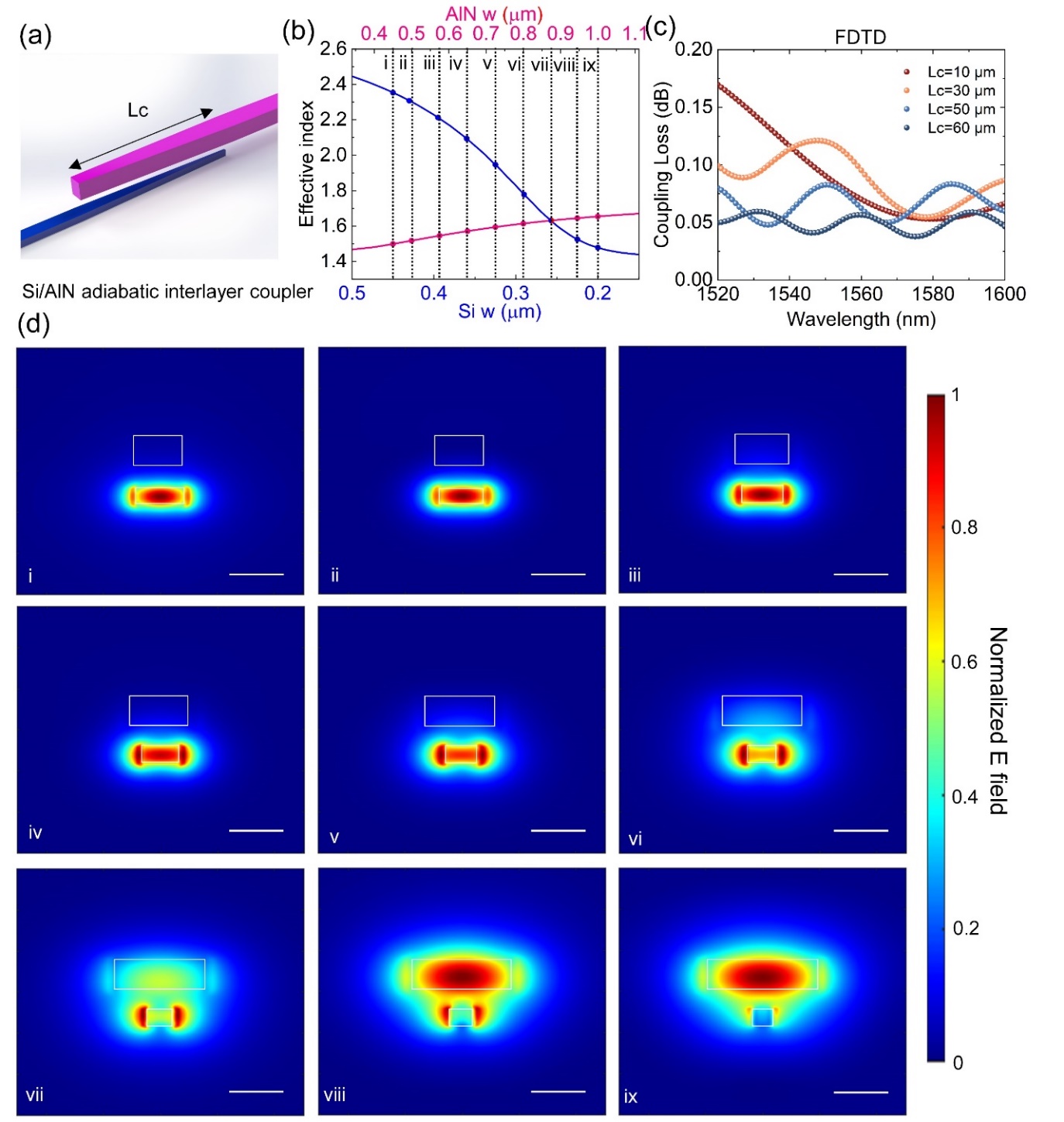


Fig. S4 Design and characterization of AlN-Si interlayer coupler. (a) schematic diagram of AlN-Si adiabatic interlayer waveguide coupler. (b) The effective index of AlN and Si waveguide at different positions of AlN/Si adiabatic interlayer waveguide coupler. (c) The FDTD simulation results in coupling loss at different coupling lengths. (d) the mode profile (electric field) of different positions of AlN/Si adiabatic interlayer waveguide coupler. 9 different positions i-ix is labeled in b with related widths in AlN and Si waveguide


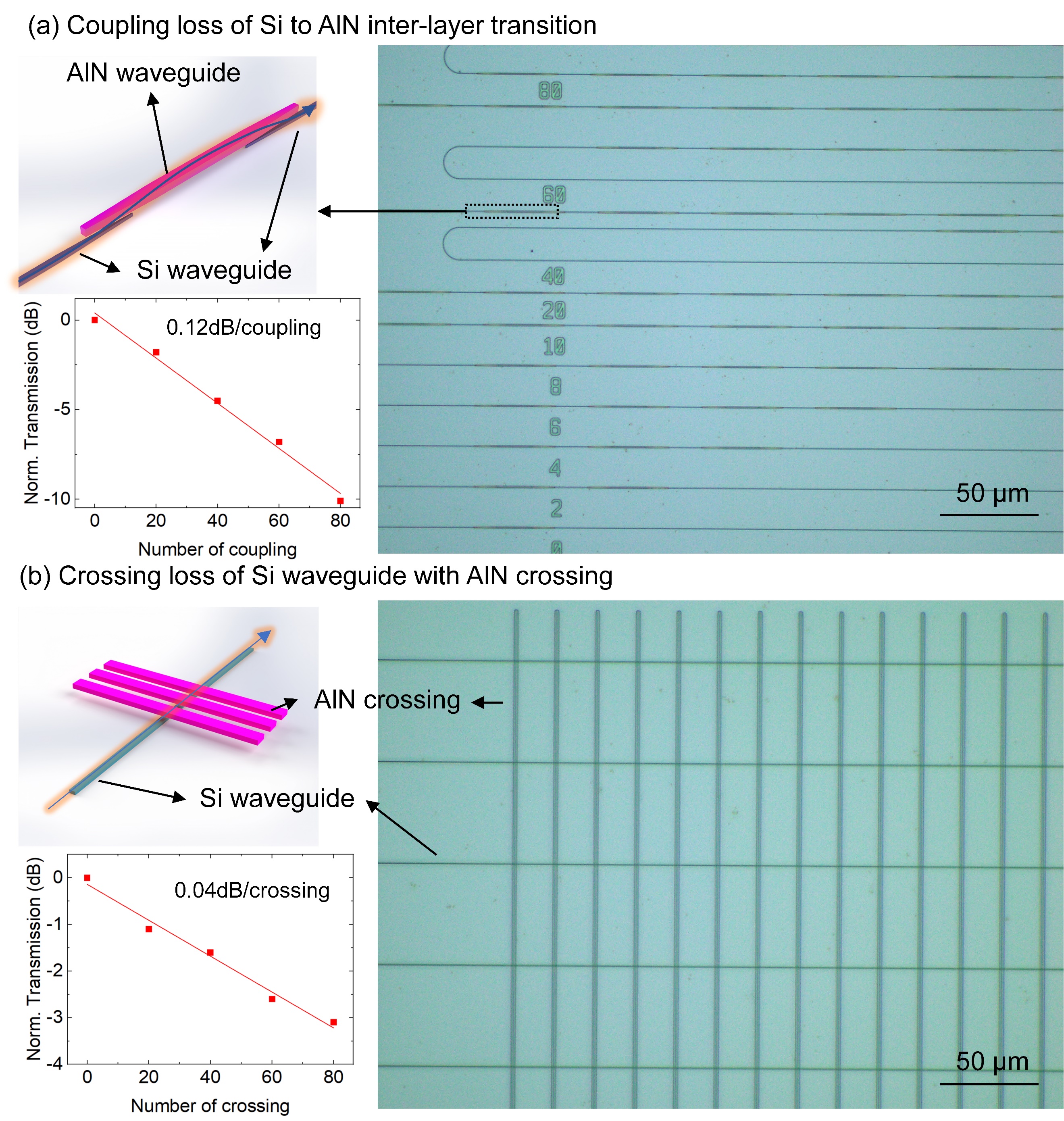


Fig. S5 Characterization of coupling loss and crossing loss of AlN/Si photonic platform. (a) Device schematic diagram, optical image, and measurement results of AlN/Si adiabatic interlayer coupler. (b) Device schematic diagram, optical image, and measurement results of Si waveguide with AlN crossing


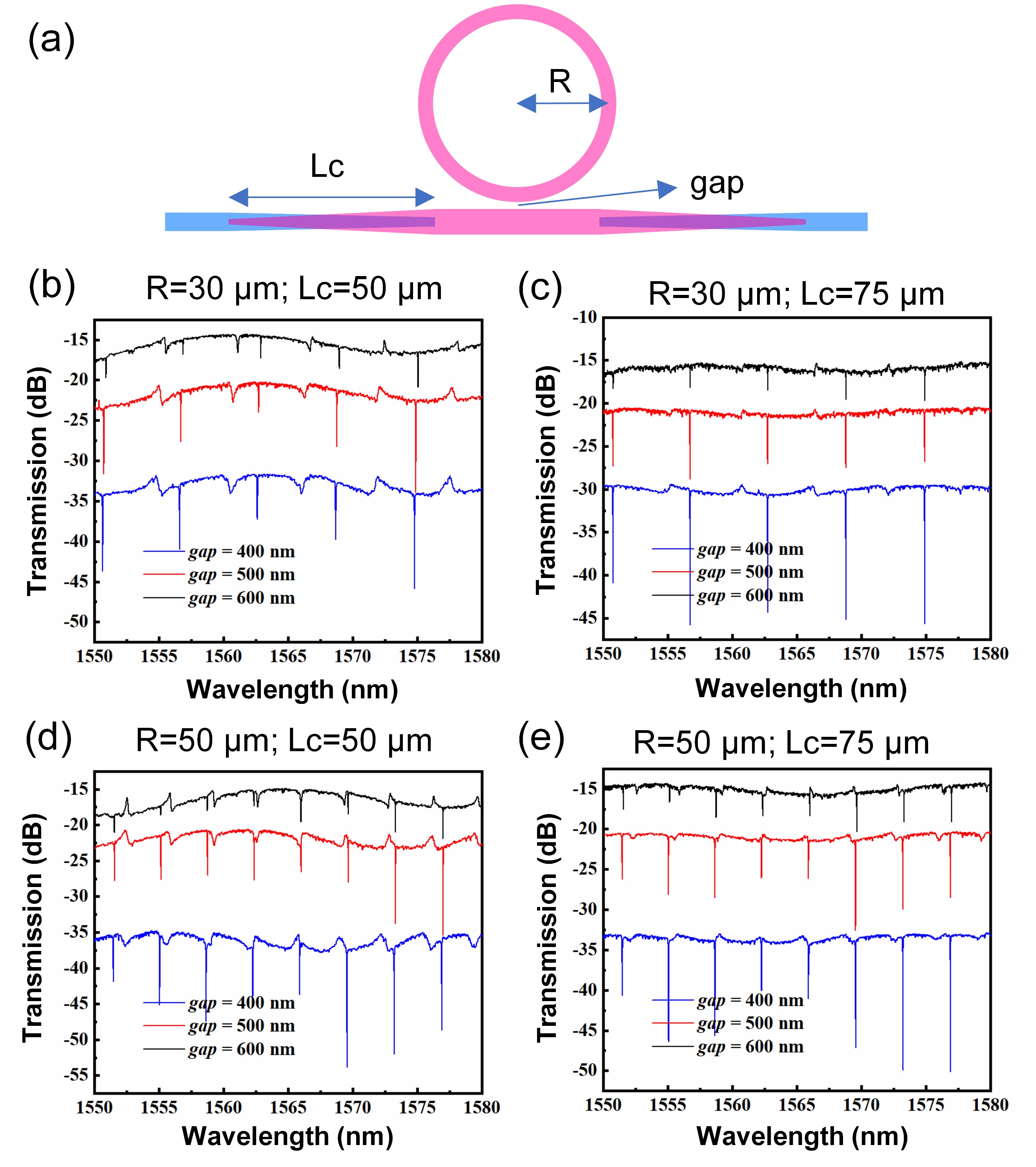


Fig. S6 The passive devices characterization of different AlN/Si bilayer MRRs. (a) schematic diagram of AlN/Si bilayer MRRs with detailed parameters of dimensions. (b-e) Measurement spectra of AlN/Si bilayer MRRs with different *R*, *Lc,* and *gaps*


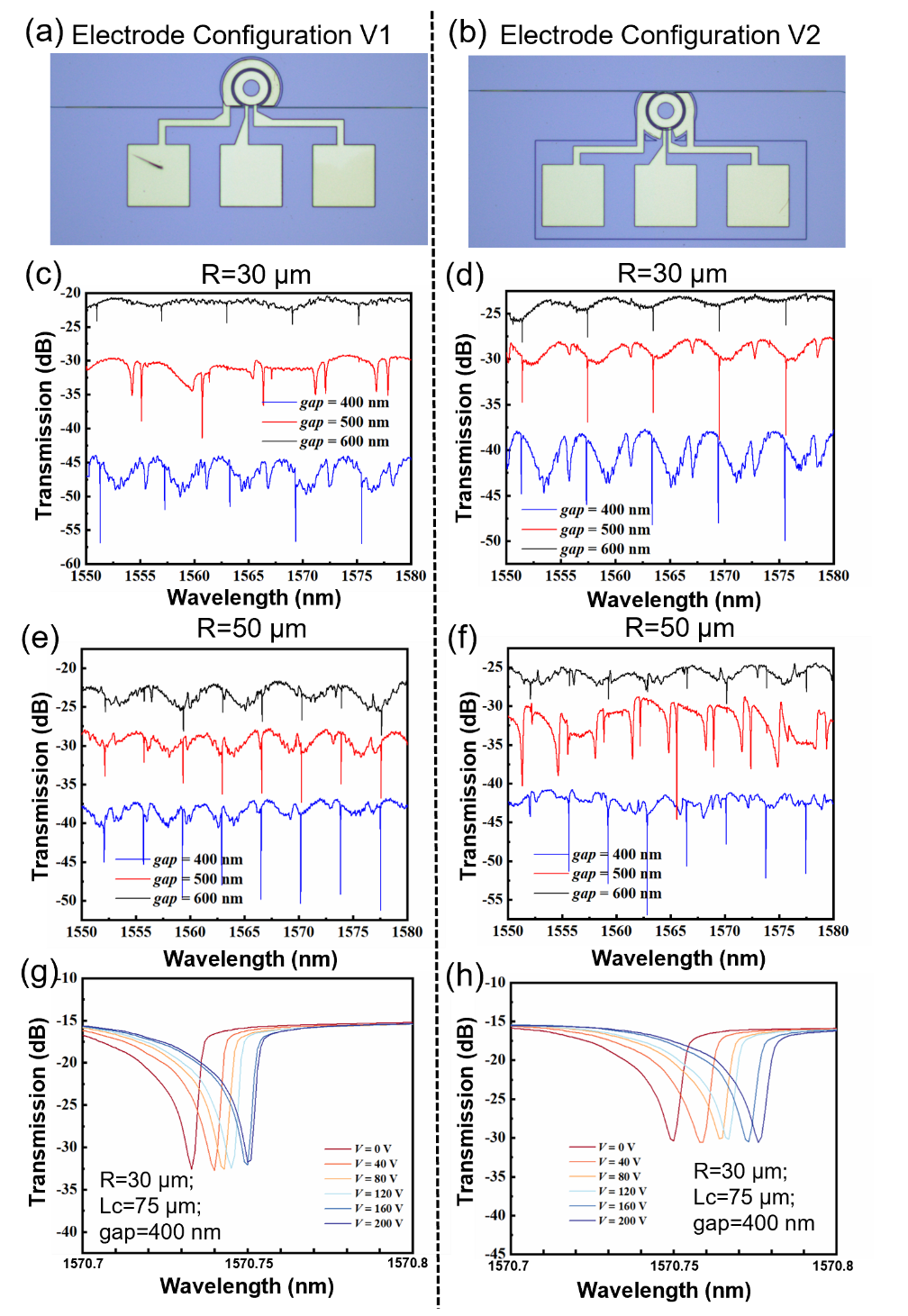


Fig. S7 The active devices characterization of different AlN/Si bilayer MRRs. (a, b) Optical microscope image of AlN/Si bilayer MRRs with different electrode configurations. (c-d) the spectra of AlN/Si bilayer MRRs with a radius of 30 µm and different electrode configurations V1 (c) and V2 (d). (e-f) the spectra of AlN/Si bilayer MRRs with a radius of 30 µm and different electrode configurations V1 (e) and V2 (f). (g-h) The tuning performance of AlN EOMs with different electrode configurations V1 (g) and V2 (h)


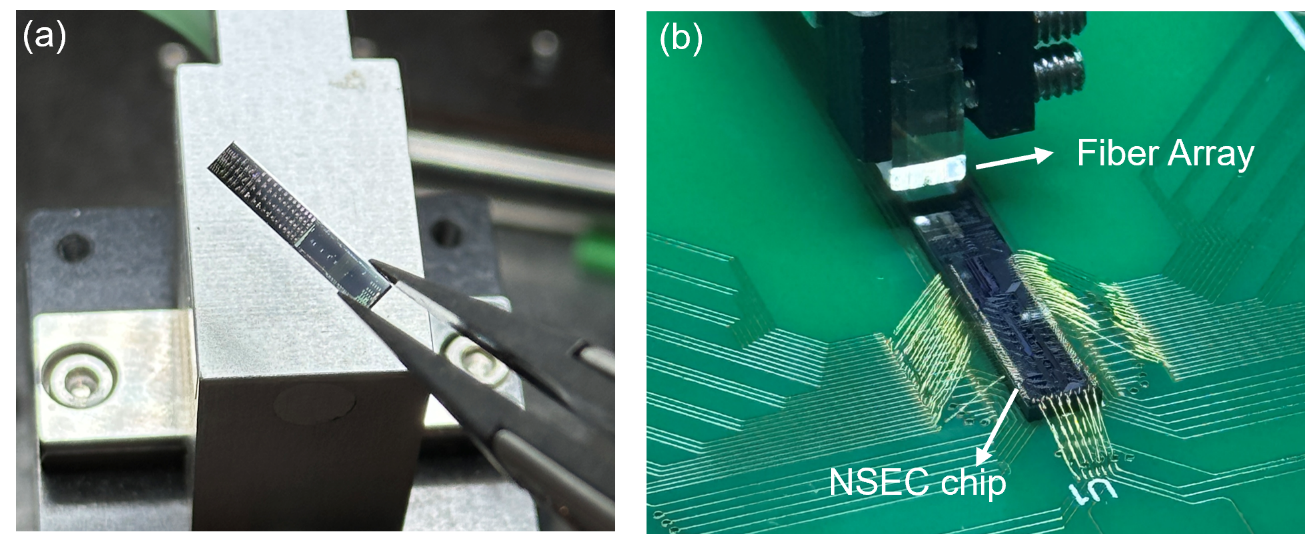
Fig. S8 The NSEC chip and integration setup. (a) The photo of photonic near-sensor computing chip die. (b) the NESC chip with wire bonding with PCB and alignment with fiber array


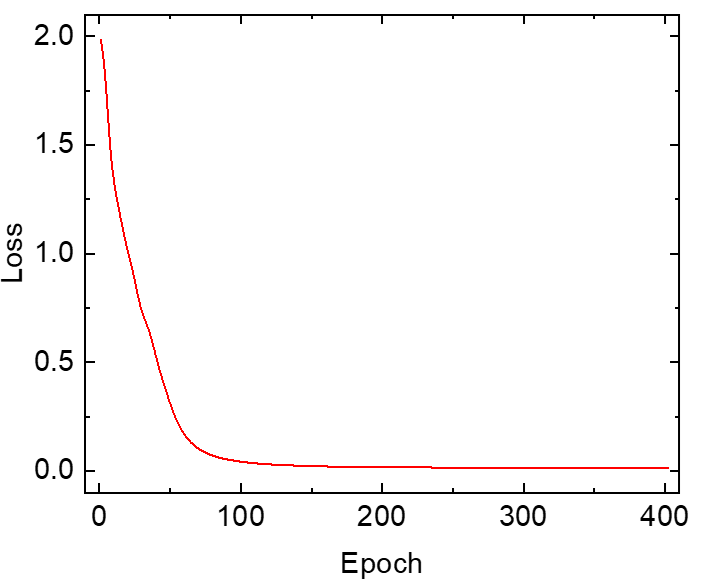


**Fig. S9** Training loss curve of the NSEC system. The loss decreases rapidly in the initial epochs and converges to near zero after approximately 100 epochs, demonstrating effective model training and convergence for photonic neural network-based AI inference

Table S1 The benchmark table of Photonic AI Chips

| **Work** | **Photonic platform** | **Photonic devices** | **Feature extraction** | | **AI processing** | | **Array size** | **AI applications and accuracy** |
| --- | --- | --- | --- | --- | --- | --- | --- | --- |
|  |  |  | **Tuning mechanism** | **Mathematic operation** | **Weighting mechanism** | **Neural Networks** |  |  |
| Integrated photonic tensor core [S1] | Si | Cross-bar array with VOAs | PCM memory | Convolution | N.A. (Done by computer) | | 4×4 | MNIST image classification, 95.3% |
| PTFP [S2] | Si | MRRs & ODLs | Si TO | Convolution | N.A. (Done by computer) | | 3×3 | Video  action recognition, 97.9% |
| IDNN [S3] | Si | Diffractive cells & MZIs | Si TO | FT & IFT | N.A. (Done by computer) | | 1×10 | Image classification, 89.4% (MNIST), 81.4% (Fashion MNIST) |
| Microcomb photonic processing unit [S4] | AlGaAs & Si* | AlGaAs Comb; Si MRRs & ODLs | Si TO | Convolution | N.A. (Done by computer) | | 1×8 (2×2 kernels) | MNIST image classification, 96.6% |
| In-memory photonic dot-product engine [S5] | Si | VOAs | PCM memory | Convolution | N.A. (Done by computer) | | 1×4 (2×2 kernels) | MNIST image classification, 87% |
| OCPU [S6] | SiN | MMIs & ODLs | SiN TO | Convolution | N.A. (Done by computer) | | 1×4 (2×2 kernels) | MNIST image classification, 92.71% |
| High-dimensional photonic tensor core [S7] | Si | Cross-bar array with VOAs &MZIs | PCM memory & Si TO | Convolution | N.A. (Done by computer) | | 3×3 | ECG classification, 93.5% |
| PDNN [S8] | Si | VOAs & MRRs | N.A. | | Si PIN | Fully-connected | 5×6 | Image classification 93.8% (2 classes), 89.8% (4 classes) |
| Lithography-free integrated photonic processor [S9] | InGaAsP | MMI | N.A. | | InGaAsP QW | Fully-connected | 8×4 | Vowel classification, 93.8% |
| DONN-I1/I3/M3 [S10] | Si | SWUs & MZIs | N.A. | | Si TO | DONN | 4×3 | MNIST image classification, 86% |
| L^2^ONN [S11] | Si | SWUs | N.A. | | Si TO | Sparse  optical neuron | 16×4 | Fashion MNIST image classification, 82.6% |
| NSEC (our work) | AlN/Si multi-layer | AlN MRRs & Si MZIs | AlN EO | Integration | Si TO | Fully-connected | 4×4 | Multimodality sensing, Gesture recognition, 96.77% (13 classes), Gait analysis, 98.31% (7 classes) |

*Demonstrated in separate chips. ^#^VOA: variable optical attenuator; PCM: phase change material; MNIST: modified National Institute of Standards and Technology database; PTFP: photonic tensor flow processer; MRR: micro-ring resonator; ODL: optical delay line; TO: thermo-optic; IDNN: integrated diffractive neural networks; MZI: Mach–Zehnder interferometer; FT: Fourier transform; IFT: inverse Fourier transform; OCPU: optical convolution processing unit; MMI: multimode interference; ECG: electrocardiogram; PDNN: photonic deep neural network ; PIN: P-doped–intrinsic–N-doped; QW: quantum well; DONN: diffractive optical neural network; SWU: Subwavelength unit; L^2^ONN: lifelong-learning optical neural network; NSEC: near-sensor edge computing.

**Table S2** The benchmark table of In/Near-sensor computing solutions

| **Work** | **Sensory devices** | **AI inference devices** | **Latency** | **Energy** | **AI applications and accuracy** |
| --- | --- | --- | --- | --- | --- |
| Memristor-based adaptive neuromorphic  Perception [S12] | Piezoresistive tactile sensor and camera | Memristor array | 1 ms | / | Tactile and visual perception, 94% |
| Retrainable neuromorphic biosensor [S13] | Ion-selective organic  electrochemical transistors (IS-OECT) | Electrochemical random-access memories (EC-RAM) | ~ms | / | Sweat analysis, 100% (2 class) |
| In-sensor adaptive wearable biosensing system [S14] | Surface electromyography (s-EMG) | System-on-a-chip field-programmable gate array (SoC FPGA) | 250 ms | 2.44 μJ (Sensing)  4.39 μJ (Classification) | Hand gesture recognition, 92.87% (21 classes) |
| In-sensor reservoir computing system [S15] | Photo-synapse | Memristor array | 25 ms | 0.5 nJ (Sensing) | Fingerprint recognition, >90% |
| All-analog chip  combining electronic and light computing (ACCEL) [S16] | Photodiode array | Optical analog computing (OAC) and Electronic analog  computing (EAC) | 72 ns | 11.8 nJ (Sensing)  3.2 nJ (Classification) | Image and video classification, 85.5%(Fashion-MNIST), 82.0% (3-class ImageNet), 92.6%,  (time-lapse video) |
| In-sensor spiking neural networks [S17] | Programmable WSe_2_  photodiode | Programmable WSe_2_  photodiode | 5 μs | / | Motion  recognition, 92% |
| NSEC (our work) | TENG gloves and socks | AlN/Si waveguide | ~10 ns | Self-powered (Sensing)  <0.34 pJ (Classification) | Multimodality sensing, Gesture recognition, 96.77% (13 classes), Gait analysis, 98.31% (7 classes) |

Supplementary References

1. J. Feldmann, N. Youngblood, M. Karpov, H. Gehring, X. Li, et al., Parallel convolutional processing using an integrated photonic tensor core. Nature **589**, 52 (2021). <https://doi.org/10.1038/s41586-020-03070-1> .
2. S. Xu, J. Wang, S. Yi, W. Zou, High-order tensor flow processing using integrated photonic circuits. Nat. Commun. **13**, 7970 (2022). <https://doi.org/10.1038/s41467-022-35723-2>
3. H.H. Zhu, J. Zou, H. Zhang, Y.Z. Shi, S.B. Luo, et al., Space-efficient optical computing with an integrated chip diffractive neural network. Nat. Commun. **13**, 1044 (2022). <https://doi.org/10.1038/s41467-022-28702-0>
4. B. Bai, Q. Yang, H. Shu, L. Chang, F. Yang, et al., Microcomb-based integrated photonic processing unit. Nat. Commun. **14**, (2023). <https://doi.org/10.1038/s41467-022-35506-9>
5. W. Zhou, B. Dong, N. Farmakidis, X. Li, N. Youngblood, et al., In-memory photonic dot-product engine with electrically programmable weight banks. Nat. Commun. **14**, 1 (2023). <https://doi.org/10.1038/s41467-023-38473-x>
6. X. Meng, G. Zhang, N. Shi, G. Li, J. Azaña, et al., Compact optical convolution processing unit based on multimode interference. Nat. Commun. **14**, 1 (2023). <https://doi.org/10.1038/s41467-023-38786-x>
7. B. Dong, S. Aggarwal, W. Zhou, U.E. Ali, N. Farmakidis, et al., Higher-dimensional processing using a photonic tensor core with continuous-time data. Nat. Photonics **17**, 1080 (2023). <https://doi.org/10.1038/s41566-023-01313-x>
8. F. Ashtiani, A. J. Geers, F. Aflatouni, An on-chip photonic deep neural network for image classification. Nature **606**, 501 (2022). <https://doi.org/10.1038/s41586-022-04714-0>
9. T. Wu, M. Menarini, Z. Gao, L. Feng, Lithography-free reconfigurable integrated photonic processor. Nat. Photonics **17**, 710 (2023). <https://doi.org/10.1038/s41566-023-01205-0>
10. T. Fu, Y. Zang, Y. Huang, Z. Du, H. Huang, et al., Photonic machine learning with on-chip diffractive optics. Nat. Commun. **14**, 70 (2023). <https://doi.org/10.1038/s41467-022-35772-7>
11. Y. Cheng, J. Zhang, T. Zhou, Y. Wang, Z. Xu, et al., Photonic neuromorphic architecture for tens-of-task lifelong learning. Light Sci. Appl. **13**, 56 (2024). <https://doi.org/10.1038/s41377-024-01395-4>
12. S. Wang, S. Gao, C. Tang, E. Occhipinti, C. Li, et al., Memristor-based adaptive neuromorphic perception in unstructured environments. Nat. Commun. 1 (2024). <https://doi.org/10.1038/s41467-024-48908-8>
13. E. R. W. van Doremaele, X. Ji, J. Rivnay, and Y. van de Burgt, A retrainable neuromorphic biosensor for on-chip learning and classification. Nat. Electron. **6**, 765 (2023). <https://doi.org/10.1038/s41928-023-01020-z>
14. A. Moin, A. Zhou, A. Rahimi, A. Menon, S. Benatti, et al., A wearable biosensing system with in-sensor adaptive machine learning for hand gesture recognition. Nat. Electron. **4**, 54 (2021). <https://doi.org/10.1038/s41928-020-00510-8>
15. Z. Zhang, X. Zhao, X. Zhang, X. Hou, X. Ma, et al., In-sensor reservoir computing system for latent fingerprint recognition with deep ultraviolet photo-synapses and memristor array. Nat. Commun. **13**, 6590 (2022). <https://doi.org/10.1038/s41467-022-34230-8>
16. Y. Chen, M. Nazhamaiti, H. Xu, Y. Meng, T. Zhou, et al., All-analog photoelectronic chip for high-speed vision tasks. Nature **623**, 48 (2023). <https://doi.org/10.1038/s41586-023-06558-8>
17. Y. Zhou, J. Fu, Z. Chen, F. Zhuge, Y. Wang, et al., Computational event-driven vision sensors for in-sensor spiking neural networks. Nat. Electron. **6**, 870 (2023). <https://doi.org/10.1038/s41928-023-01055-2>
